# Microbial Diversity of Bovine Mastitic Milk as Described by Pyrosequencing of Metagenomic 16s rDNA

**DOI:** 10.1371/journal.pone.0047671

**Published:** 2012-10-17

**Authors:** Georgios Oikonomou, Vinicius Silva Machado, Carlos Santisteban, Ynte Hein Schukken, Rodrigo Carvalho Bicalho

**Affiliations:** Department of Population Medicine and Diagnostic Sciences, College of Veterinary Medicine, Cornell University, Ithaca, New York, United States of America; Auburn University, United States of America

## Abstract

Dairy cow mastitis is an important disease in the dairy industry. Different microbial species have been identified as causative agents in mastitis, and are traditionally diagnosed by bacterial culture. The objective of this study was to use metagenomic pyrosequencing of bacterial 16S rRNA genes to investigate bacterial DNA diversity in milk samples of mastitic and healthy dairy cows and compare the results with those obtained by classical bacterial culture. One hundred and thirty-six milk samples were collected from cows showing signs of mastitis and used for microbiological culture. Additionally, 20 milk samples were collected from healthy quarters. Bacterial DNA was isolated from the same milk samples and the 16S rRNA genes were individually amplified and pyrosequenced. Discriminant analysis showed that the groups of samples that were most clearly different from the rest and thus easily discriminated were the normal milk samples from healthy cows and those characterised by culture as *Trueperella pyogenes and Streptococcus* spp. The mastitis pathogens identified by culture were generally among the most frequent organisms detected by pyrosequencing, and in some cases (*Escherichia coli*, *Klebsiella* spp. and *Streptococcus uberis* mastitis) the single most prevalent microorganism. *Trueperella pyogenes* sequences were the second most prevalent sequences in mastitis cases diagnosed as *Trueperella pyogenes* by culture, *Streptococcus dysgalactiae* sequences were the second most prevalent sequences in mastitis cases diagnosed as *Streptococcus dysgalactiae* by culture, and *Staphyloccocus aureus* sequences were the third most prevalent in mastitis cases diagnosed as *Staphylococcus aureus* by culture. In samples that were aerobic culture negative, pyrosequencing identified DNA of bacteria that are known to cause mastitis, DNA of bacteria that are known pathogens but have so far not been associated with mastitis, and DNA of bacteria that are currently not known to be pathogens. A possible role of anaerobic pathogens in bovine mastitis is also suggested.

## Introduction

Dairy cow mastitis is arguably the most important disease for the dairy industry worldwide, causing economic losses due to reduced milk production, discarded milk, premature culling, and antibiotic usage. Clinical mastitis is also a serious animal welfare issue as it is associated with pain and reduced well-being of the affected animals [1].

Identification of the bacteria responsible for mastitis is an important component of eventual clinical resolution of the disease. Currently, bacterial culture is the gold standard method for identification of mastitis-causing microorganisms. However, limitations of classical bacterial culture such as a delay of 24–48 hours to obtain results, or the fact that in approximately 25% of milk samples from clinical mastitis cases bacteria are not detected in conventional culture [2] have spurred investigations of culture-independent, molecular techniques for mastitis diagnosis. Methods such as real-time PCR [3], multiplex PCR (mPCR) [4], denaturing gradient gel electrophoresis (DGGE) PCR [5], and PCR-single-strand conformation polymorphism (SSCP) [6] are now being used to identify bacterial DNA in milk samples. Apart from addressing the above mentioned limitation in classical culture, molecular techniques also have the potential to offer some insight in the microbial communities present in milk [7].

Sequencing and analysis of hypervariable regions within the 16S rRNA gene can provide relatively rapid and cost-effective methods for assessing bacterial diversity and abundance and may be useful for pathogen discovery and identification [8]. Barcoded pyrosequencing on the Genome Sequencer FLX/454 Life Sciences platform, enable a dramatic increase in throughput via parallel in-depth analysis of many samples with limited sample processing and lower costs. In a recent publication, Hunt et al. [7] used barcoded pyrosequencing to characterize the diversity of bacterial communities in human milk samples and showed that this technique identified a much greater diversity of bacteria in milk than what has previously been reported in culture-independent studies. Additionally, Bhatt et al. [9] used shotgun pyrosequencing to analyse the milk microbiome of Kankrej, Gir (Bos indicus) and crossbred (Bos Taurus × Bos indicus) cattle with subclinical mastitis. To the best of our knowledge, barcoded pyrosequencing of the 16S rRNA gene has not yet been used to investigate milk samples from mastitic Holstein dairy cows.

The aim of this study was to evaluate metagenomic pyrosequencing of the 16S rRNA gene for investigation of bacterial diversity in milk samples of mastitic dairy cows.

## Results

### Diagnosis of Mastitis Aetiology by Bacterial Culture

Based on bacterial culture of milk samples from 136 mastitic dairy cows, 16 cases were diagnosed as infected with *Streptococcus uberis*, 17 as *Escherichia coli*, 11 as *Klebsiella* spp., 17 as *Staphylococcus aureus*, 20 as *Staphylococcus* spp., 17 as *Streptococcus dysgalactiae*, 2 as *Streptococcus* spp., 3 as *Trueperella pyogenes*, and 33 samples were characterized as culture negative. The 20 milk samples obtained from healthy cows were also found to be culture negative.

### Classification of Bacterial Species in Milk from Mastitic Cows Based on 16S rRNA Gene Sequences

The same 156 milk samples were also subjected to metagenomic pyrosequencing of bacterial 16S rRNA genes. Pyrosequencing produced 283,713 sequences; their size ranged from 36 to 1102 bp. The sequences that were finally analysed by the RDP classifier (after trimming and quality control) were 240,524; their size ranged from 250 to 504 bp. To facilitate comparison, the sequencing results have been grouped according to the culture-based mastitis diagnosis; sequences derived from the milk samples obtained from healthy cows were also grouped separately.

**Figure 1:**
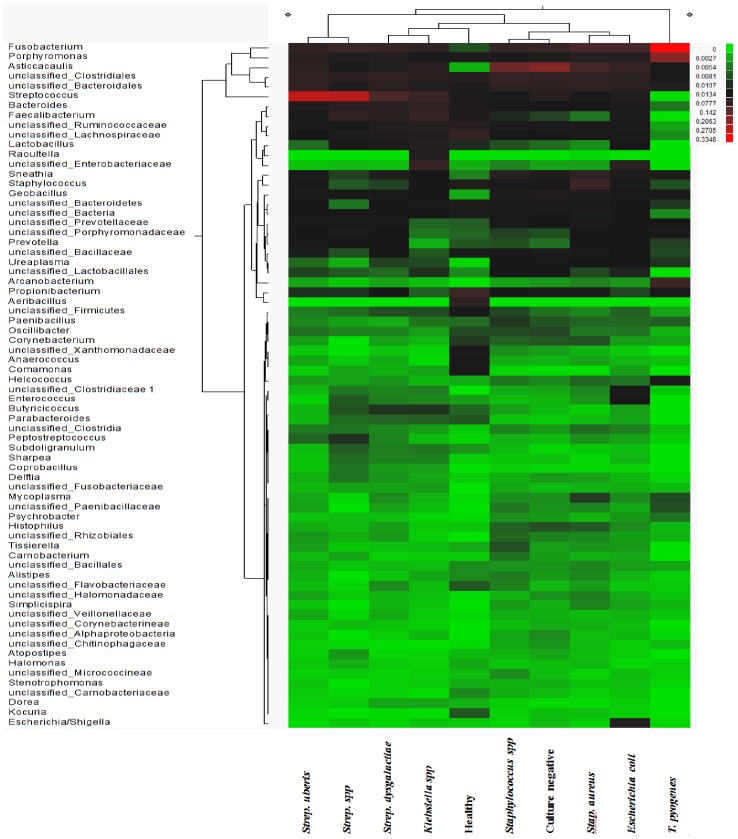
Heat map illustrating different genera mean prevalence by culture-based diagnosis. The heat map was generated by hierarchical cluster analysis of different genera mean prevalence using the statistical software JMP (SAS InstituteInc., Cary, NC).

Information regarding mean genera prevalence in each group of samples, derived from classification of the samples’ sequences by the RDP classifier, is illustrated with the use of a heat map (Figure 1).

**Figure 2:**
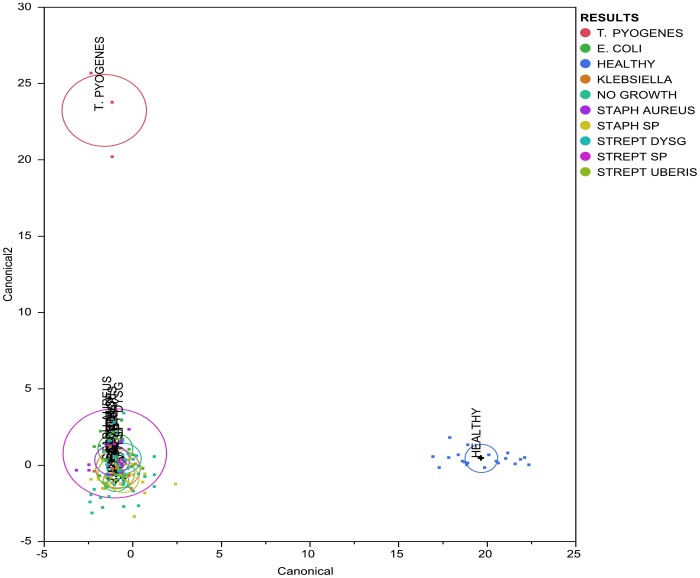
Discriminant analysis of mastitic and normal milk samples microbiome by culture-based diagnosis. Discriminant analysis was performed in JMP Pro (SAS Institute Inc. North Carolina) using the bacterial genus prevalence in each sample as covariates and the culture-based diagnosis as the categorical variable.

**Figure 3:**
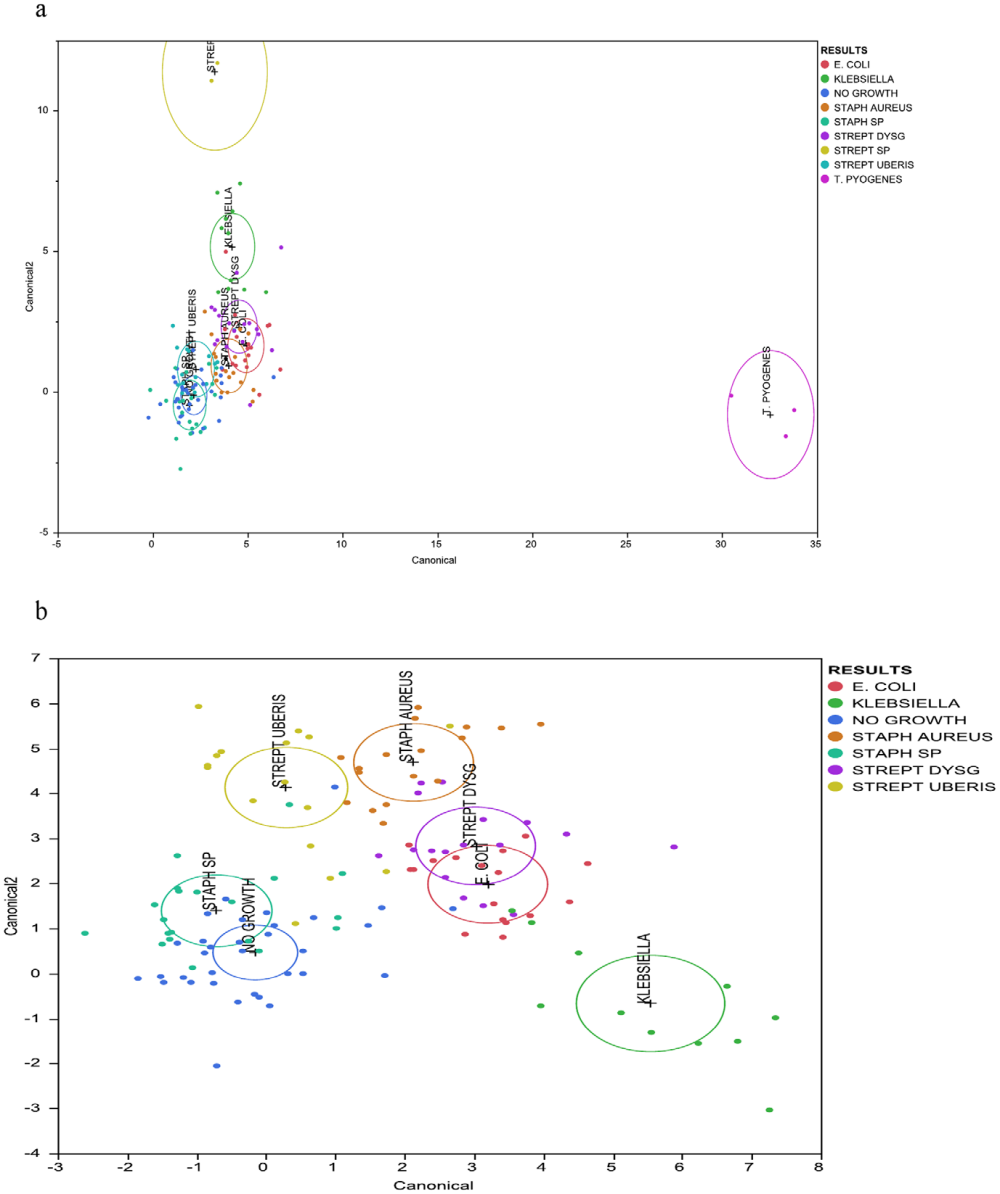
Discriminant analysis of mastitic milk samples microbiome by culture-based diagnosis. Discriminant analysis was performed in JMP Pro (SAS Institute Inc. North Carolina) using the bacterial genus prevalence in each sample as covariates and the culture-based diagnosis as the categorical variable. Results derived from discriminant analysis that was performed using all groups of mastitic samples are presented in Figure 3a. Additionally, discriminant analysis was performed using only groups of samples characterized by culture as *Streptococcus uberis, Escherichia coli*, *Klebsiella* spp., *Staphylococcus aureus*, *Staphylococcus* spp., *Streptococcus dysgalactiae*, and as culture negative (no growth). Results from this analysis are presented in Figure 3b.

**Figure 4:**
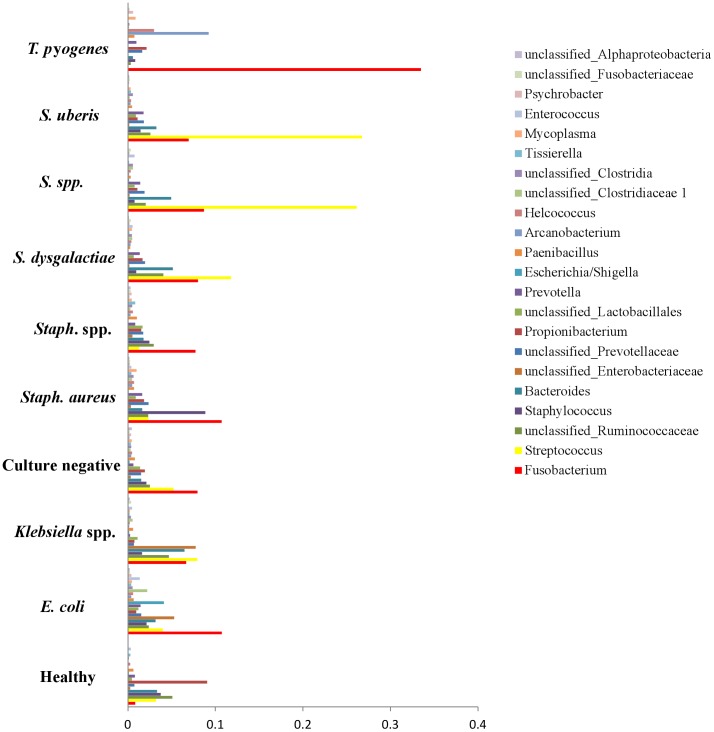
Mean prevalence, by different culture-based diagnosis, of bacterial genera that were found to be significant for the discriminant analysis of mastitic milk samples.

Results derived from the discriminant analysis that used bacterial genera prevalence as covariates and the culture-based diagnosis as the categorical variable are presented in Figure 2 and 3. Results derived from discriminant analysis that was performed using all groups of samples are presented in Figure 2. Additionally, discriminant analysis was performed using only groups of samples from mastitic cows (Figure 3a) and using only groups of samples characterized by culture as *Streptococcus uberis, Escherichia coli*, *Klebsiella* spp., *Staphylococcus aureus*, *Staphylococcus* spp., *Streptococcus dysgalactiae*, and as culture negative. Results derived from this analysis are presented in Figure 3b. The mean prevalence, by different culture-based diagnosis, of bacterial genera that were found to be significant for the discriminant analysis that used only groups of samples from mastitic cows is presented in Figure 4.

Tables S1–S10 in the supplemental material, list the species-level information (with GenBank accession numbers and percentages of identity match) for each different group of samples. Prevalence in these tables is defined as the number of sequences that were found to belong to each specific Operational Taxonomic Unit (OTU) out of the total number of sequences analyzed for each group of samples. The mastitis causative agent diagnosed by culture was generally among the organisms most frequently detected by pyrosequencing, and in three cases *(Escherichia coli*, *Klebsiella* spp. and *Streptococcus uberis* mastitis) it was the single most prevalent microorganism. *Trueperella pyogenes* sequences were the second most prevalent sequences in mastitis cases diagnosed as *T. pyogenes* by culture, *Streptococcus dysgalactiae* sequences were the second most prevalent sequences in mastitis cases diagnosed as *S. dysgalactiae* by culture, and *Staphyloccocus aureus* sequences were the third most prevalent in mastitis cases diagnosed as *S. aureus* by culture.

**Figure 5:**
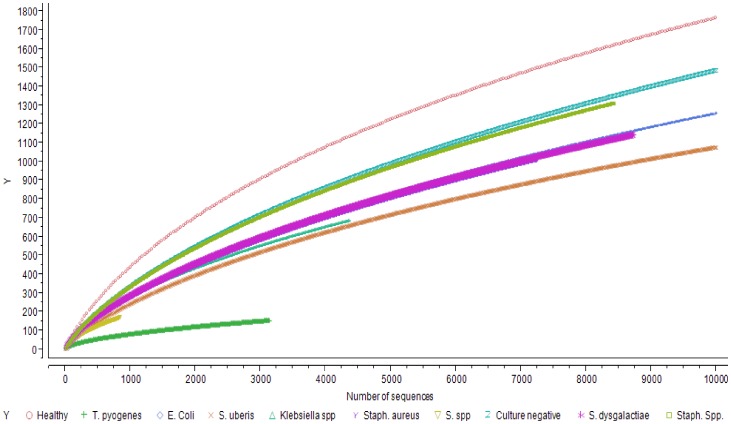
Rarefaction curves of different groups of samples for a cutoff value of 0.03.

Shannon and Chao1 indices estimates for different distance cut-off values (0.01, 0.03 and 0.05) are presented in Table 1. Rarefaction curves for each different group of samples are presented in Figure 5.

**Figure 6:**
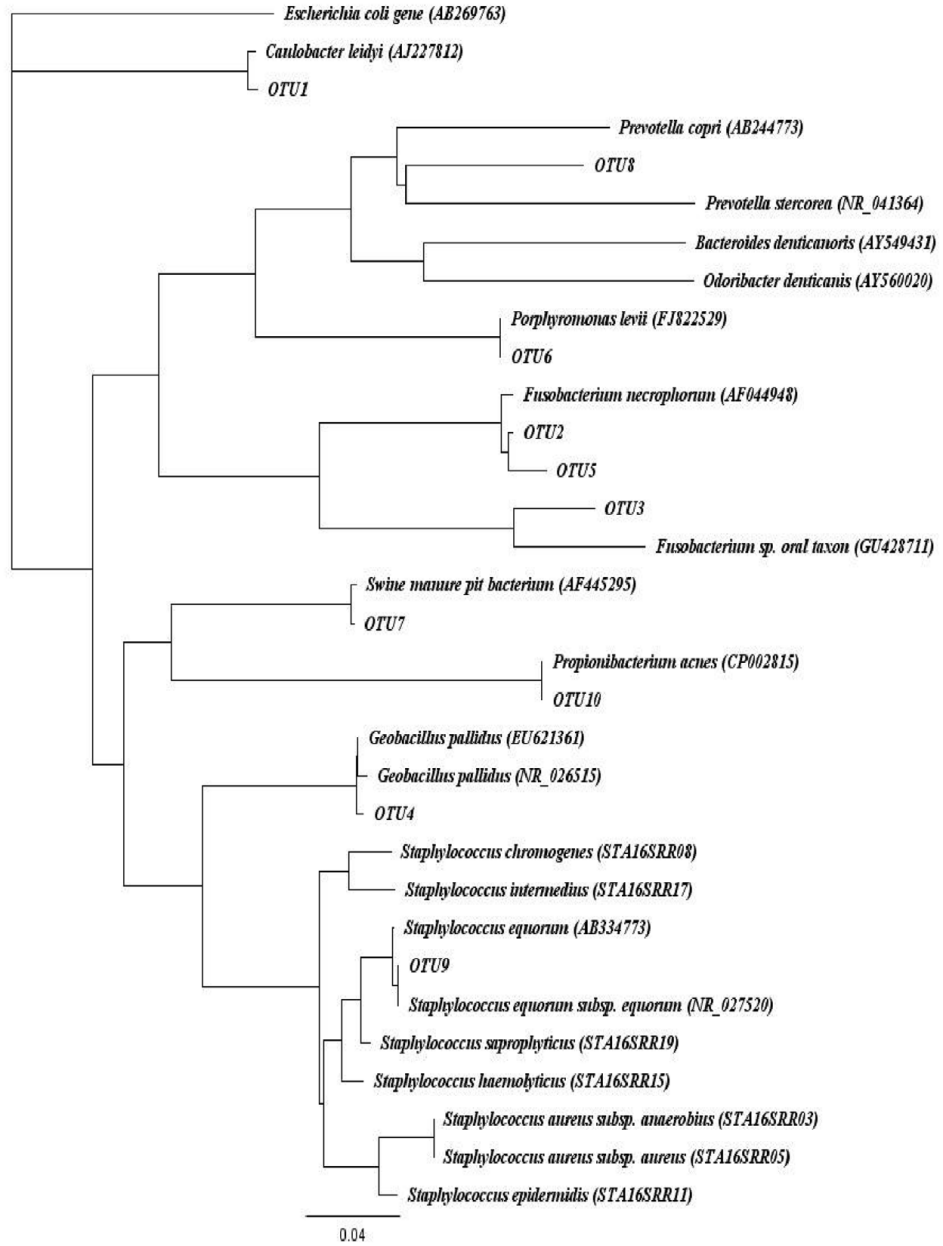
Phylogenetic tree of the ten most predominant sequences (OTU1–OTU10, also presented in supplemented material Table 6) of samples characterized as *Staphylococcus* spp. mastitis. *Escherichia coli* served as outgroup. GenBank accession numbers are indicated in parentheses.

Since species level results regarding samples diagnosed as *Staphylococcus* spp. by classical culture were unexpected, a phylogenetic tree of the ten most predominant sequences in the samples characterized as *Staphylococcus* spp. mastitis was built to provide additional information and is presented in Figure 6.

**Table 1:** Chao1 and Shannon indices for different cutoff distances (0.01, 0.03 and 0.05) and for different group of samples.

	Distancecutoff	Number of samples/sequences analyzed	Clusters	Chao1	Shannon Index (H′)
	0.01		75	144.46	3.30
*Trueperella pyogenes*	0.03	3/304	49	82.55	2.97
	0.05		44	74.30	2.84
	0.01		597	2311.78	5.29
*Escherichia coli*	0.03	20/1473	343	1207.39	4.51
	0.05		283	846.89	4.23
	0.01		304	909.36	4.94
*Klebsiella pneumoniae*	0.03	11/674	188	422.00	4.19
	0.05		163	279.91	4.04
	0.01		1176	3399.11	5.64
*Culture negative*	0.03	33/3008	591	1337.23	4.52
	0.05		472	916.54	4.35
	0.01		719	2453.41	5.36
*Staphylococcus aureus*	0.03	17/1862	386	892.95	4.48
	0.05		312	609.56	4.28
	0.01		676	2374.15	5.33
*Staphylococcus* spp.	0.03	20/1555	411	1015.20	4.55
	0.05		339	627.02	4.37
	0.01		489	1754.64	5.13
*Streptococcus dysgalactiae*	0.03	17/1263	280	599.22	4.42
	0.05		237	480.29	4.21
	0.01		57	140.25	3.43
*Streptococcus* spp.	0.03	2/151	46	104.00	3.11
	0.05		43	118.60	2.90
	0.01		461	1601.00	4.17
*Streptococcus uberis*	0.03	16/1487	273	681.49	3.57
	0.05		235	468.93	3.45
	0.01		641	2096.00	5.52
*Normal milk samples*	0.03	20/1307	427	864.27	4.98
	0.05		376	704.91	4.85

**Figure 7:**
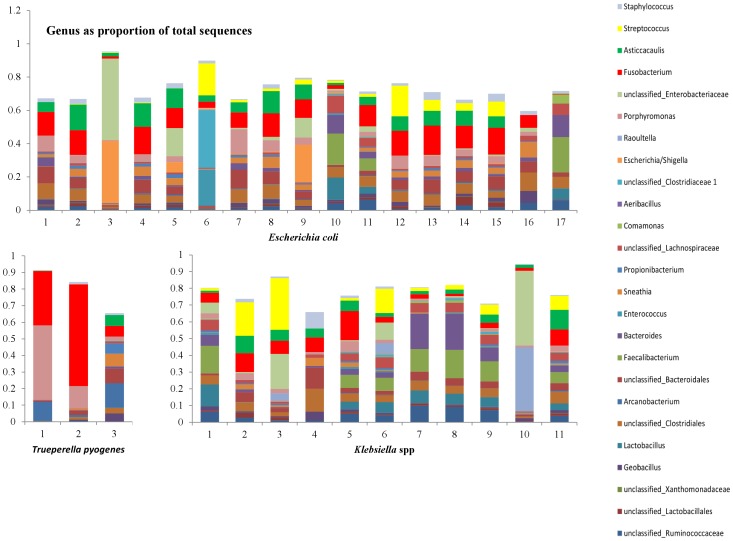
Distribution of the genera as a percentage of the total number of identified 16S sequences in individual samples that were classified either as *Escherichia coli* (top, n = 17), or *Trueperella pyogenes* (bottom left, n = 3), or *Klebsiella* spp. (bottom right, n = 11) in classical bacteriology.

**Figure 8:**
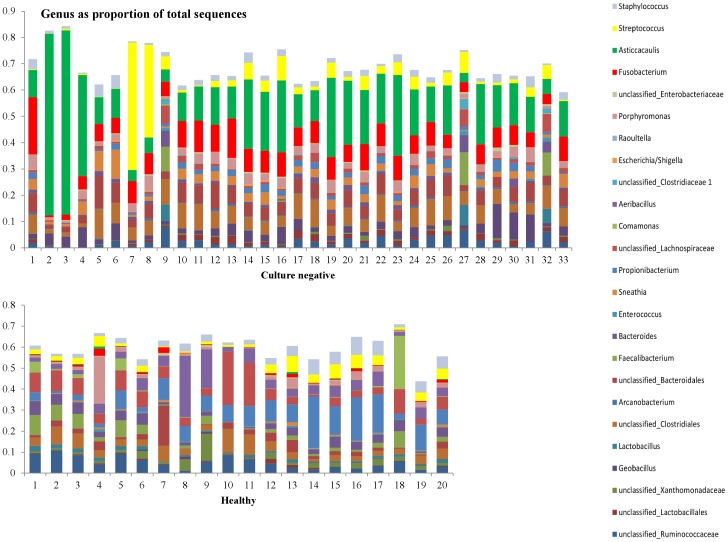
Distribution of the genera as a percentage of the total number of identified 16S sequences in individual samples that were classified either as culture negative in classical bacteriology (top, n = 33), or were obtained from healthy cows (bottom, n = 20).

**Figure 9:**
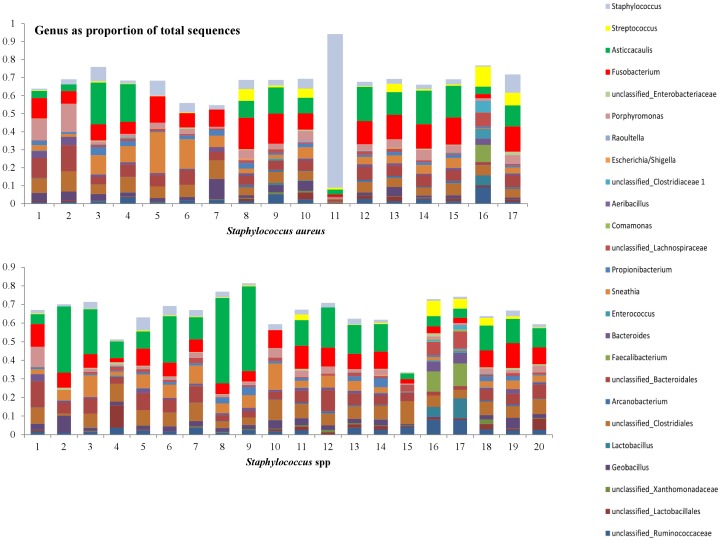
Distribution of the genera as a percentage of the total number of identified 16S sequences in individual samples that were classified either as *Staphylococcus aureus* (top, n = 17), or as *Staphylococcus* spp. (bottom, n = 20) in classical bacteriology.

**Figure 10:**
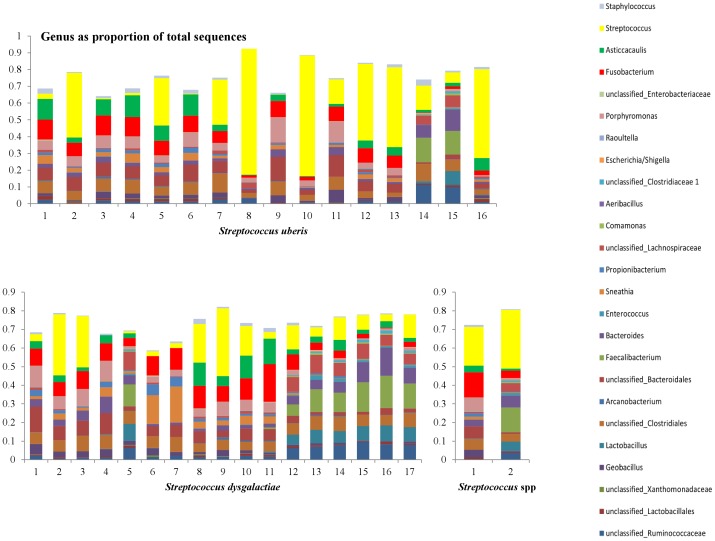
Distribution of the genera as a percentage of the total number of identified 16S sequences in individual samples that were classified either as *Streptococcus uberis* (top, n = 16), or as *Streptococcus dysgalactiae* (bottom left, n = 17), or as *Streptococcus* spp. (bottom right, n = 2) in classical bacteriology.

In Figures 7–10, the genera that contribute to the sequences identified in individual samples are shown. We show here the genera to contribute at least 10% to any single sample from the 156 samples. Two samples in the culture negative collection (Figure 8, samples 7 and 8) showed a high proportion of *Streptococcus* spp. Upon further evaluation of the sequences at species level, the majority of these sequences were from the *Streptococcus uberis* species.

## Discussion

The bacteria diagnosed by aerobic culture generally corresponded to the most frequent bacterial sequences detected by pyrosequencing, as shown in Tables S1–S9 of the supplemental material. However, along with this confirmation came important additional information.

As it is shown in the heatmap presented in Figure 1, sequences classified by the RDP classifier as *Escherichia/Shigella* spp., *Raoultella* spp. and *Arcanobacterium* spp., were mainly prevalent in the groups of samples characterized by culture as *Escherichia coli, Klebsiella* spp. and *Trueperella pyogenes* respectively. *Streptococcus* spp. showed higher prevalence in the groups characterized as *Streptococcus* spp., *Streptococcus uberis* and *Streptococcus dysgalactiae*. However, *Streptococcus* spp. was also prevalent in all other groups of samples, even in the milk samples derived from healthy cows. A similar observation was made for *Staphylococcus* spp. Interestingly, Hunt et al. [7] reported that *Staphylococcus* spp. and *Streptococcus* spp. were part of the core microbiome of non-mastitic human milk samples.

Discriminant analysis showed that the microbiota of samples derived from healthy cows was clearly different from the microbiota of the mastitic samples. Discrimant analysis performed using only the mastitic samples showed that the groups of samples that were most clearly different from the rest and thus easily discriminated were the ones characterised as *Trueperella pyogenes and Streptococcus spp.* These were also the groups of samples that showed the lowest diversity having the lowest Shannon and Chao1 indices. *Streptococcus dysgalactiae* samples were found to group together with *Escherichia coli* samples, and culture negative samples grouped with *Staphylococcus* spp. samples. When *T. pyogenes* and *Streptococcus* spp. samples were not included in the discriminant analysis, *Staphylococcus* spp. samples grouped with culture negative samples and *Streptococcus dysgalactiae* samples with *E. coli samples*, while the remaining groups were well discriminated. Sun et al. [10] already reported that the application of discriminant analysis enables the derivation of quantitative disease-associated microbial signatures and the description of microbial community structure in a detailed way. This notion seems to be, at least partially, confirmed by results presented here. Additionally, as it is shown in Figure 4, not all genera that were found to be highly prevalent in most samples were also significant for the discriminant analysis. While *Fusobacterium* spp. (prevalent in most samples) was highly significant for the discriminant analysis, *Asticcacaulis* spp. (a genus also shown to be prevalent in most samples, and highly prevalent in samples that were culture negative) was not.

We were able to identify high numbers of anaerobic bacterial sequences in all mastitis cases, regardless of the culture-based diagnosis. The role of anaerobic bacteria (*Fusobacterium necrophorum*, *Bacteroides* spp., *Porphyromonas levii*) and their synergistic action with *Trueperella pyogenes* (formerly known as *Arcanobacterium* or *Actinomyces pyogenes*) in the aetiology of summer mastitis has been established [11]. Milk samples from cases that were diagnosed as *T. pyogenes* mastitis in this study were also found to have a high prevalence of DNA sequences from *F. necrophorum* subsp. *funduliforme*. DNA from *F. necrophorum* and other anaerobic bacteria that are known pathogens (e.g. *P. levii* [12]) was detected in most of the mastitic milk samples regardless of the culture-based diagnosis. On the other hand, *F. necrophorum* sequences were practically absent in the 20 samples that were derived from healthy, low somatic cell count quarters, while *Porphyromonas* spp. sequences were detected but in low prevalence comparing to their prevalence in the mastitic samples.

Prevalence studies lack the ability to show a proper time order of cause and effect, experimental challenge studies with known quantities of bacteria would be able to provide such causal arguments. Despite these limitations, a possible role of certain anaerobic bacteria as opportunistic pathogens can be speculated based on the results presented here. Our group recently showed a high prevalence of anaerobic bacteria such as *Fusobacterium necrophorum* and *Porphyromonas levii* in the uteri of metritic cows, and these bacteria were absent from the uterine lumen of healthy animals [13]. In another recent publication we showed that *Escherichia coli* infection is actually creating a suitable intrauterine environment for the establishment and persistence of uterine infection by *F. necrophorum* [14]. Results presented here allow us to suggest that the role of anaerobic bacteria in mastitis has probably been underestimated due to the difficulties related to their culturing. The role of *F. necrophorum* as an opportunistic pathogen deriving either from the cow’s rumen (formation of liver abscesses) or from the environment (interdigital necrobacillosis) is already known [15]. Additionally, Bhatt et al. [9] used shotgun pyrosequencing to analyse milk samples derived from cows with subclinical mastitis and also reported the presence of anaerobic bacteria (e.g. *Fusobacteriales*, *Bacteroidales*).

In the 33 samples that were identified by classical aerobic culture techniques as culture negative, pyrosequencing was able to detect sequences from bacteria that are known to cause bovine mastitis; this included *Streptococcus uberis*, *Trueperella pyogenes,* and *Escherichia coli*. As it is shown in Figure 8, *Streptococci* were prevalent in almost all the culture negative samples. Two of the culture negative samples showed a very high proportion of *Streptococci*, further evaluation of the individual sequences showed a predominance of *S. uberis* in these samples. Koskinen et al. [3] using RT-PCR were able to detect *S. uberis* in 30, *T. pyogenes* in 15, and *E. coli* in 18 out of 180 culture negative samples from clinical mastitis. Taponen et al. [2] specifically studied samples that were culture negative in classical culture detecting S. *uberis* by RT-PCR in 10 out of 79 culture negative samples, while *T. pyogenes* and *E. coli* were detected in only 1 sample each. Bexiga et al. [16] also used RT-PCR on 51 samples derived from subclinical mastitis cases, negative in classical culture; 6 samples were found positive for *E. coli,* 1 sample was found positive for *T. pyogenes,* 1 for *S. uberis* and 1 for *S. dysgalactiae.* Pyrosequencing used approximately 100 times more the amount of milk compared to classical culture. This could result in a higher likelihood of finding pathogenic bacteria, even if the classical culture did not identify these bacteria. A simple sampling argument might explain some of the observed differences between the two methods. This is also true for the RT-PCR methods [2], where approximately 35 times the amount of milk is used for the molecular diagnostic method. It should also be mentioned that routine mastitic milk aerobic culture will only detect aerobic bacteria that are still alive and reproducing at the time of the sample analysis, while this requirement of live bacteria is not a limitation for molecular DNA based techniques.

The most prevalent bacterial sequences in the culture negative samples were from *Caulobacter leidyia* in the family Sphingomonadaceae. The same bacterium was also prevalent (in lower numbers) in most of the milk samples with a positive bacterial identification, while it was also prevalent (in even lower numbers) in samples derived from healthy cows. Since *C. leidyia* is an aquatic bacterium [17] its presence in milk samples might be a result of water contamination during the sampling process, or it may be present in high numbers in the milking parlor, where it may have opportunity to invade the udder when the cow’s defences are compromised due to mastitis. The observation that *C. leidyia’s* relative abundance is greater in certain mastitis cases, especially cases that are reported as culture negative, is interesting, although its significance will require further investigation.

There were a number of samples in this study where a mastitis pathogen was identified by culture, while pyrosequencing revealed the presence of other known mastitis pathogens. For example, in a number of samples diagnosed by bacterial culture as *Klebsiella spp*. or *Escherichia coli*, DNA of *Streptococcus uberis* was also detected by pyrosequencing. Kuang et al. [5] using DGGE PCR and Koskinen et al. [3] using RT-PCR also suggested the possibility of mixed bacterial infections in some mastitis cases that went undetected when traditional culturing technique was used. Bhatt et al. [9] also supported this assumption and suggested that subclinical mastitis is not merely caused by a single pathogenic species of bacteria, but rather by a blend of several microbes. On the other hand, presence of many different bacterial species in a milk sample is considered to be a sign of contamination in the case of classical culture. The presence of many bacterial species in quantities that are not identified by culture but are identified by pyrosequencing may also reflect an underlying contamination of small amounts of bacterial DNA during the process of sampling, sample transport and sample handling on the farm or in the laboratory.

Pyrosequencing results regarding mastitis cases diagnosed by culturing as *Streptococcus* spp. showed that the most prevalent representative sequence belonged to *Streptococcus macedonicus*. On the other hand, *S. macedonicus* sequences were not detected in the milk samples obtained from healthy cows. This microorganism was first isolated from Kasseri, a traditional Greek semi-hard cheese [18], and has not up to now been correlated with mastitis. It was reported as a causative agent in one case of human endocarditis [19].

In samples characterized by culture as *Staphylococcus* spp., *Staphylococcus equorum* was detected by pyrosequencing. *Staph. equorum* sequences were not detected in the healthy milk samples. *Staph. equorum* is a coagulase negative *Staphylococcus* and has previously been isolated from bovine mastitis cases [20], [21]. As depicted in Figure 6, our *Staph. equorum* sequences group with sequences from other species of *Staphylococci* such as *Staphylococcus chromogenes* that are also coagulase negative and generally considered to be mastitis pathogens [22]. Bexiga et al. [16] studied 32 samples identified as *Staphylococcus spp.* by classical culture and identified in 30 of these samples *Staphylococcus spp.* using RT-PCR. Surprisingly, only 1.8% of all identified sequences were associated with *Staphylococcus spp.* in this study, and these were not necessarily known dominant *Staphylococcus spp.* associated with bovine mastitis such as *Staph. chromogenes*. Results derived by discriminant analysis suggest that similarities exist between samples characterized as *Staphylococcus* spp. and culture negative samples. Evaluation of the genera distribution in individual samples showed a remarkable similarity between culture negative samples and *Staphylococcus* species. This would suggest that the identified *Staphylococcus* species in classical culture form a small proportion of total bacterial DNA in the sample, resulting apparently in the minor pathology that is typically associated with this bacterial species [23].

*Helcococcus ovis* is a gram-positive, catalase-negative coccus that is associated with endocarditis in bovines [24]. In the present study, *H. ovis* was prevalent in samples from culture negative, *Escherichia coli, Staphylococcus aureus, Staphylococcus* spp. and *Streptococcus uberis* mastitis cases. However, *H. ovis* was also prevalent in the milk samples obtained from healthy cows. Schwaiger et al. [6] recently reported for the first time the presence of this microorganism in dairy cow mastitic milk samples and cautiously suggested its possible involvement in the pathogenesis of bovine mastitis. *H. ovis* has also been isolated from a sheep with subclinical mastitis [25].

The results from this study should be treated as tentative because they cannot by themselves prove pathogenicity of bacteria identified by the presence of their DNA. The pyrosequencing method will detect dead organisms and pieces of DNA that may be irrelevant to bovine mastitis. Finally, contamination from the environment with minute quantities of bacterial DNA during sampling, transport or handling could provide results as reported here.

In conclusion, the use of metagenomic pyrosequencing of the 16S rRNA should be considered an important tool to advance our knowledge regarding the pathogenesis of bovine mastitis and could be developed as a diagnostic tool. Opportunistic anaerobic bacteria such as *Fusobacterium necrophorum* and *Porphyromonas levii* were highly prevalent in mastitic milk samples and this was not the case for the non-mastitic milk samples. Further longitudinal studies are suggested to elucidate the complex and potentially dynamic microbiology of milk in healthy cows and in cows with subclinical and clinical mastitis.

## Materials and Methods

### Sampling and Microbiological Culture

One hundred and thirty-six (136) milk samples were collected from cows with clinical or subclinical mastitis and sent to the Quality Milk Production Services (QMPS) laboratory at Cornell University for microbiological culture. For comparison purposes 20 milk samples obtained from healthy quarters from cows that had no history of mastitis and found to have a somatic cell count lower than 10,000 were also used. Samples were taken after teat ends had been disinfected with alcohol and the first streams of milk were discarded. Approximately 0.01 ml of each milk sample was inoculated using cotton swabs on trypticase soy agar plates containing 5% sheep blood and 0.1% esculin (bioMerieux, INC. Durham, NC 27704-0969 USA) and incubated aerobically at 37°C. Bacterial growth was identified after 24 and 48 h of incubation according to National Mastitis Council standards. Briefly, *Staphylococcus aureus* and *Staphylococcus* spp. were identified by haemolytic pattern and tube coagulate test. *Streptococcus dysgalactiae, Streptococcus uberis* and *Streptococcus* spp. were differentiated by presence or absence of esculin hydrolysis, Lancefield group C typing (PathoDx strep grouping latex agglutination test, Remel), and growth or growth inhibition on Bile Esculin Azide Agar (Enterococcosel™, Becton, Dickinson). *Escherichia coli* and *Klebsiella* spp. were identified using morphologic characteristics of colonies on MacConkey agar, production of indole, motility, and utilization of citrate. *Trueperella pyogenes* was identified by colonial characteristic, presence of complete hemolysis and Gram stain. No mycoplasma culture or anaerobic culture was performed on the samples. Samples that showed a conclusive culture result with a single dominant pathogen or the absence of any growth in the aerobic culture process were selected for this study.

### DNA Extraction

One millilitre of milk from the same sample that was used for bacterial culture was centrifuged for 10 min at room temperature at 13,200 rpm (16,100 rcf) in an Eppendorf 5415R centrifuge. The supernatant was discarded and the remaining pellet was resuspended in 400 µl of nuclease-free water. Isolation of genomic DNA was then performed by using a QIAamp DNA minikit (Qiagen) according to the manufacturer’s instructions, except that 400 µg of lysozyme was added to the bacterial suspension and incubated for 12 h at 56°C to maximize bacterial DNA extraction. DNA concentration and purity were evaluated by optical density using a NanoDrop ND-1000 spectrophotometer (NanoDrop Technologies, Rockland, DE, USA) at wavelengths of 230, 260 and 280 nm.

### PCR Amplification of the V1-2 Region of Bacterial 16S rRNA Genes

The 16S rRNA genes were individually amplified from each sample using a composite pair of primers containing a unique 10-base barcode, which was used to tag the PCR products from the respective samples. The forward primer was 5′-**CGTATCGCCTCCCTCGCGCCATCAG**NNNNNNNNNNTC *AGAGTTTGATCCTGGCTCAG*-3′: the bold sequence is the GS FLX Titanium Primer A, and the italicized sequence is the universal broadly conserved bacterial primer 27F. The reverse primer was 5′-**CTATGCGCCTTGCCAGCCCGCTCAG**NNNNNNNNNN CA *TGCTGCCTCCCGTAGGAGT*-3′: the bold sequence is the GS FLX Titanium Primer B, and the italicized sequence is the broad-range bacterial primer 338R. The sequence NNNNNNNNNN, which is identical in the forward and reverse primer of each pair, designates the unique 10-base barcode used to tag each PCR product. A two-base linker sequence (underlined) was inserted between the barcode and the template-specific sequence to help diminish any effect the composite primer might have on the efficiency of the amplifications. The specific pair of primers used was checked against the bovine genome with NCBI primer-BLAST [26] and was not found to anneal with bovine DNA. PCRs were carried out in triplicate 20-µl reactions containing 0.3 µM forward and reverse primers, using approximately 50 ng of template DNA and 10 µl HotStar Taq Plus Mix kit (Qiagen). A modified touchdown thermal cycling was used for amplification and consisted of initial denaturation at 95°C for 2 min, followed by 30 cycles of denaturation at 95°C for 30 sec, annealing (starting at 68°C and subsequently decreased by 2°C/2 cycles until it reached 58°C at which temperature the 20 remaining cycles were performed) for 30 sec, extension at 72°C for 60 sec, and a final extension at 72°C for 7 min. Replicate amplicons were pooled, purified with a QIAquick PCR Purification Kit (Qiagen), and visualized by electrophoresis using 1.2% (wt/vol) agarose gels stained with 0.5 µg/ml ethidium bromide before sequencing. Blank controls, in which no DNA was added to the reaction, were performed. In all cases these blank controls failed to produce visible PCR products; these samples were not analyzed further.

### Barcoded Pyrosequencing of Bacterial 16S rRNA Genes

Amplicons were quantified using the Quant-iT PicoGreen dsDNA Assay Kit (Invitrogen) and combined in equimolar ratios into a single tube with a final concentration of 16 ng/µl. Pyrosequencing of the samples was carried at the Cornell University Life Sciences Core Laboratories Center using Roche 454 GS-FLX System Titanium Chemistry.

### Sequences Library Analysis

Sorting by tag sequence, trimming and quality control of sequences that derived from pyrosequencing, were done by Geneious [27]. One mismatch was allowed in the barcode, while two mismatches were allowed in the primers. Primers were removed from the sequences, zero N’s were allowed while sequences shorter than 250 bp were also removed. The Ribosomal Database Project (RDP) Classifier at the RDP’s Pyrosequencing Pipeline was used to assign 16S rRNA gene sequences of each sample to the new phylogenetically consistent higher-order bacterial taxonomy, using an 80% confidence threshold, providing information regarding different genera prevalence in each sample [28]. Different genera prevalence in each sample derived from this analysis were used as covariates in a discriminant analysis that was performed in JMP Pro (SAS Institute Inc. North Carolina). The culture-based diagnosis was used as the categorical variable in this analysis. A heat map was generated by hierarchical cluster analysis of different genera using the statistical software JMP (SAS Institute Inc., Cary, NC).

To facilitate a detailed (species level) analysis of the sequences, the following steps were followed: 200 sequences from each sample with the same culture-based diagnosis were randomly selected, using the random number function of Excel, and used to create a new FASTA sequence file. This file was then processed through the RDP pyrosequencing pipeline [29]. Specifically, the file was first uploaded in the pipeline initial processor that trimmed the 16S primers and filtered out additional sequences of low-quality. The produced file was uploaded in the RDP’s aligner, which aligns the sequences using the INFERNAL aligner, a Stochastic Context Free Grammar (SCFG)-based, secondary-structure aware aligner [30], and then processed by the complete linkage clustering tool (that clustered the aligned sequences in OTU). Finally, the dereplicate function was used to created one representative sequence for each OTU. Eventually, a new file of representative sequences was created. The Basic Local Alignment Search Tool (BLASTn algorithm) from the National Center for Biotechnology Information (NCBI) web pages (http://www. ncbi.nlm.nih.gov/BLAST/) was then used to examine the nucleotide collection (EMBL/GenBank/DDBJ/PDB) databases for sequences with high similarity to these representative sequences [31].

The cluster file that was obtained from the above described process was subsequently used for the evaluation of the samples richness and diversity through the estimation of Shannon and Chao1 indices, again using the RDP pyrosequencing pipeline [29]. The Shannon index is a nonparametric diversity index that combines estimates of richness (the total number of OTUs) and evenness (the relative abundance of OTUs). For example, communities with one dominant species have a low index, whereas communities with a more even distribution have a higher index. Chao1 is a nonparametric estimator of the minimum richness (number of OTUs) and is based on the number of rare OTUs (singletons and doublets) within a sample.

The above described process was also followed using all sequences from each group of samples until a cluster file was obtained for each group of samples. This file was subsequently used to obtain rarefaction curves for each group of samples, again using the RDP pyrosequencing pipeline [29].

To study the evolutionary relationships among the ten most predominant sequences from samples characterized as *Staphylococcus spp.* mastitis, the sequences obtained were imported into Geneious software and aligned to other 16S rRNA gene sequences using ClustalW. The alignment was further manually corrected, and calculation of the phylogenetic trees was based on these sequence alignments using the neighbor-joining algorithm [32]. Evolutionary distances were computed using the Jukes-Cantor method [33].

## Supporting Information

Table S1 **Species level information (with GenBank Accession number, and identity match) for the predominant representative sequences in samples characterized as culture negative.**
(DOCX)

Table S2 **Species level information (with GenBank Accession number, and identity match) for the predominant representative sequences in samples characterized as Trueperella pyogenes mastitis.**
(DOCX)

Table S3 **Species level information (with GenBank Accession number, and identity match) for the predominant representative sequences in samples characterized as Escherichia coli mastitis.**
(DOCX)

Table S4 **Species level information (with GenBank Accession number, and identity match) for the predominant representative sequences in samples characterized as Klebsiella pneumoniae mastitis.**
(DOCX)

Table S5 **Species level information (with GenBank Accession number, and identity match) for the predominant representative sequences in samples characterized as Staphylococcus aureus mastitis.**
(DOCX)

Table S6 **Species level information (with GenBank Accession number, and identity match) for the predominant representative sequences in samples characterized as Staphylococcus spp. Mastitis.**
(DOCX)

Table S7 **Species level information (with GenBank Accession number, and identity match) for the predominant representative sequences in samples characterized as Streptococcus dysgalactiae mastitis.**
(DOCX)

Table S8 **Species level information (with GenBank Accession number, and identity match) for the predominant representative sequences in samples characterized as Streptococcus spp. Mastitis.**
(DOCX)

Table S9 **Species level information (with GenBank Accession number, and identity match) for the predominant representative sequences in samples characterized as Streptococcus uberis mastitis.**
(DOCX)

Table S10 **Species level information (with GenBank Accession number, and identity match) for the predominant representative sequences in samples obtained from healthy cows and had a low SCC.**
(DOCX)
