# Supplementary material for: Microbial Diversity of Bovine Mastitic Milk as Described by Pyrosequencing of Metagenomic 16s rDNA
Source: PLoS One. 2012 Oct 17;7(10):e47671. doi: 10.1371/journal.pone.0047671 (PMC3474744; doi:10.1371/journal.pone.0047671)
Supplement: Table S1 — Species level information (with GenBank Accession number, and identity match) for the predominant representative sequences in samples characterized as culture negative. (DOCX) [file pone.0047671.s001.docx]

| Species | Accession No | Prevalence | Identity (%) |
| --- | --- | --- | --- |
| *Caulobacter leidyia* | GQ891704 | 20.55 | 98.9 |
| *Uncultured Fusobacteria* | EF704825 | 5.49 | 100 |
| *Geobacillus pallidus* | FJ808716 | 5.09 | 100 |
| *Streptococcus uberis* | HQ326694 | 4.82 | 100 |
| *Uncultured bacterium* | JF643239 | 3.32 | 100 |
| *Propionibacterium acnes* | CP002409 | 2.76 | 100 |
| *Uncultured bacterium* | EU289919 | 2.46 | 100 |
| *Porphyromonas levii* | AB547664 | 2.09 | 100 |
| *Uncultured Porphyromonas* | HM754526 | 1.46 | 99 |
| *Staphylococcus equorum* | AB334773 | 1.40 | 99.7 |
| *Swine manure* | AF445295 | 1.33 | 99.7 |
| *Bacteroides heparinolyticus* | GQ422742 | 1.30 | 100 |
| *Ureaplasma diversum* | NR_025878 | 1.23 | 99 |
| *Paenibacillus borealis* | HM563046 | 1.03 | 99.4 |
| *Uncultured Porphyromonas* | HM754526 | 1.00 | 100 |
| *Uncultured bacterium* | EU290110 | 0.96 | 99.7 |
| *Uncultured Prevotella* | GU905978 | 0.96 | 99.2 |
| *Uncultured bacterium* | AM183009 | 0.70 | 95.6 |
| *Prevotella spp.* | FJ848548 | 0.70 | 100 |
| *Uncultured bacterium* | EU290098 | 0.66 | 100 |
| *Histophilus somni* | [AB176902.1](http://www.ncbi.nlm.nih.gov/nucleotide/62122464?report=genbank&log$=nucltop&blast_rank=1&RID=BAMM9CZF013) | 0.66 | 99 |
| *Uncultured bacterium* | AB107461 | 0.63 | 99.3 |
| *Bacillus spp.* | FR749853 | 0.63 | 100 |
| *Uncultured bacterium* | EF205694 | 0.60 | 100 |
| *Ochrobactrum pseudogrignonense* | FJ859687 | 0.57 | 99.6 |
| *Helcococcus ovis* | NR_027228 | 0.53 | 99.7 |
| *Corynebacterium falsenii* | AF537594 | 0.53 | 100 |
| *Uncultured bacterium* | HM316969 | 0.47 | 99.7 |
| *Trueperella pyogenes* | JN578133 | 0.43 | 100 |
| *Uncultured bacterium* | GU601118 | 0.40 | 100 |
| *Delftia spp.* | CP002735 | 0.40 | 100 |
| *Escherichia coli* | CP001671 | 0.37 | 100 |
| *Uncultured Ruminococcaceae* | EU794142 | 0.37 | 99.7 |
| *Mycoplasma bovigenitalium* | AY121098 | 0.37 | 99.7 |
| *Xanthomonas campestris* | CP002789 | 0.33 | 100 |
| *Uncultured bacterium* | HM257593 | 0.33 | 95.1 |
| *Uncultured Bacteroidetes* | FM252970 | 0.33 | 100 |
| *Brevibacillus parabrevis* | JN315628 | 0.33 | 99.7 |
| *Uncultured Staphylococcus* | JN082690 | 0.33 | 100 |
