# Supplementary material for: Microbial Diversity of Bovine Mastitic Milk as Described by Pyrosequencing of Metagenomic 16s rDNA
Source: PLoS One. 2012 Oct 17;7(10):e47671. doi: 10.1371/journal.pone.0047671 (PMC3474744; doi:10.1371/journal.pone.0047671)
Supplement: Table S2 — Species level information (with GenBank Accession number, and identity match) for the predominant representative sequences in samples characterized as Trueperella pyogenes mastitis. (DOCX) [file pone.0047671.s002.docx]

| Species | Accession No | Prevalence | Identity (%) |
| --- | --- | --- | --- |
| *Fusobacterium necrophorum subsp. Funduliforme* | [AB525413.1](http://www.ncbi.nlm.nih.gov/nucleotide/261228522?report=genbank&log$=nucltop&blast_rank=3&RID=BAMM9CZF013) | 21.05 | 100 |
| *Uncultured bacterium* | [EU290118.1](http://www.ncbi.nlm.nih.gov/nucleotide/167595709?report=genbank&log$=nucltop&blast_rank=1&RID=BAMM9CZF013) | 17.76 | 100 |
| *Uncultured bacterium* | [EU290137.1](http://www.ncbi.nlm.nih.gov/nucleotide/167595728?report=genbank&log$=nucltop&blast_rank=1&RID=BAMM9CZF013) | 13.16 | 99 |
| ***Trueperella pyogenes*** | [JN578141.1](http://www.ncbi.nlm.nih.gov/nucleotide/345847796?report=genbank&log$=nucltop&blast_rank=1&RID=BAMM9CZF013) | 11.20 | 100 |
| *Uncultured bacterium* | [GQ467006.1](http://www.ncbi.nlm.nih.gov/nucleotide/258550009?report=genbank&log$=nucltop&blast_rank=1&RID=BAMM9CZF013) | 4.28 | 99 |
| *Uncultured bacterium* | [JF643239.1](http://www.ncbi.nlm.nih.gov/nucleotide/342078424?report=genbank&log$=nucltop&blast_rank=1&RID=BAMM9CZF013) | 3.29 | 100 |
| *Porphyromonas sp.* | [FJ848565.1](http://www.ncbi.nlm.nih.gov/nucleotide/225733546?report=genbank&log$=nucltop&blast_rank=2&RID=BAMM9CZF013) | 2.63 | 99 |
| *Uncultured alpha proteobacterium* | [EU810967.1](http://www.ncbi.nlm.nih.gov/nucleotide/217038624?report=genbank&log$=nucltop&blast_rank=6&RID=BAMM9CZF013) | 2.30 | 100 |
| *Ureaplasma diversum* | [NR_025878.1](http://www.ncbi.nlm.nih.gov/nucleotide/219846288?report=genbank&log$=nucltop&blast_rank=1&RID=BAMM9CZF013) | 2.30 | 99 |
| *Staphylococcus equorum* | [AB334773.1](http://www.ncbi.nlm.nih.gov/nucleotide/154127058?report=genbank&log$=nucltop&blast_rank=2&RID=BAMM9CZF013) | 1.97 | 100 |
| *Mycoplasma bovigenitalium* | [AY121109.1](http://www.ncbi.nlm.nih.gov/nucleotide/22122026?report=genbank&log$=nucltop&blast_rank=1&RID=BAMM9CZF013) | 1.64 | 100 |
| *Geobacillus pallidus* | [HM030740.1](http://www.ncbi.nlm.nih.gov/nucleotide/295853594?report=genbank&log$=nucltop&blast_rank=4&RID=BAMM9CZF013) | 1.64 | 99 |
| *Uncultured Prevotella spp.* | [GU905978.1](http://www.ncbi.nlm.nih.gov/nucleotide/294613820?report=genbank&log$=nucltop&blast_rank=2&RID=BAMM9CZF013) | 1.32 | 99 |
| *Paenibacillus caespitis* | [AM745263.1](http://www.ncbi.nlm.nih.gov/nucleotide/150246971?report=genbank&log$=nucltop&blast_rank=1&RID=BAMM9CZF013) | 1.32 | 98 |
| *Porphyromonas levii* | [FJ822532.1](http://www.ncbi.nlm.nih.gov/nucleotide/225795241?report=genbank&log$=nucltop&blast_rank=5&RID=BAMM9CZF013) | 1.32 | 100 |
| *Bacteroides heparinolyticus* | [GQ422742.1](http://www.ncbi.nlm.nih.gov/nucleotide/257480655?report=genbank&log$=nucltop&blast_rank=1&RID=BAMM9CZF013) | 0.99 | 100 |
| *Uncultured bacterium* | [JF212531.1](http://www.ncbi.nlm.nih.gov/nucleotide/322197936?report=genbank&log$=nucltop&blast_rank=1&RID=BAMM9CZF013) | 0.66 | 100 |
| *Paenibacillaceae bacterium* | [FM173657.1](http://www.ncbi.nlm.nih.gov/nucleotide/190714870?report=genbank&log$=nucltop&blast_rank=1&RID=BAMM9CZF013) | 0.66 | 99 |
| *Prevotella spp.* | [FJ848548.1](http://www.ncbi.nlm.nih.gov/nucleotide/225733529?report=genbank&log$=nucltop&blast_rank=5&RID=BAMM9CZF013) | 0.66 | 100 |
| *Uncultured bacterium* | [EU466510.1](http://www.ncbi.nlm.nih.gov/nucleotide/169281985?report=genbank&log$=nucltop&blast_rank=1&RID=BAMM9CZF013) | 0.66 | 98 |
| *Uncultured bacterium* | [AM183009.1](http://www.ncbi.nlm.nih.gov/nucleotide/157690463?report=genbank&log$=nucltop&blast_rank=1&RID=BAMM9CZF013) | 0.66 | 94 |
| *Uncultured bacterium* | [JF532102.1](http://www.ncbi.nlm.nih.gov/nucleotide/341967287?report=genbank&log$=nucltop&blast_rank=1&RID=BAMM9CZF013) | 0.66 | 86 |
| *Bacteroides pyogenes* | [AB542769.1](http://www.ncbi.nlm.nih.gov/nucleotide/284925068?report=genbank&log$=nucltop&blast_rank=2&RID=BAMM9CZF013) | 0.66 | 99 |
| *Uncultured bacterium* | [DQ791376.1](http://www.ncbi.nlm.nih.gov/nucleotide/110704603?report=genbank&log$=nucltop&blast_rank=1&RID=BAMM9CZF013) | 0.66 | 99 |
| *Uncultured bacterium* | [GU608976.1](http://www.ncbi.nlm.nih.gov/nucleotide/290595569?report=genbank&log$=nucltop&blast_rank=1&RID=BAMM9CZF013) | 0.33 | 97 |
| *Uncultured Peptostreptococcus spp.* | [EU029258.1](http://www.ncbi.nlm.nih.gov/nucleotide/154757025?report=genbank&log$=nucltop&blast_rank=1&RID=BAMM9CZF013) | 0.33 | 99 |
| *Pseudomonas fluorescens* | [AM779082.1](http://www.ncbi.nlm.nih.gov/nucleotide/158147918?report=genbank&log$=nucltop&blast_rank=1&RID=BAMM9CZF013) | 0.33 | 99 |
| *Uncultured bacterium* | [EU472381.1](http://www.ncbi.nlm.nih.gov/nucleotide/169287856?report=genbank&log$=nucltop&blast_rank=1&RID=BAMM9CZF013) | 0.33 | 99 |
| *Uncultured bacterium* | [AB107469.1](http://www.ncbi.nlm.nih.gov/nucleotide/68988911?report=genbank&log$=nucltop&blast_rank=1&RID=BAMM9CZF013) | 0.33 | 100 |
| *Uncultured bacterium* | [FJ685469.1](http://www.ncbi.nlm.nih.gov/nucleotide/223689766?report=genbank&log$=nucltop&blast_rank=1&RID=BAMM9CZF013) | 0.33 | 99 |
| *Uncultured bacterium* | [JN021854.1](http://www.ncbi.nlm.nih.gov/nucleotide/339521312?report=genbank&log$=nucltop&blast_rank=2&RID=BAMM9CZF013) | 0.33 | 99 |
| *Paenibacillus spp.* | [AB505863.1](http://www.ncbi.nlm.nih.gov/nucleotide/239937267?report=genbank&log$=nucltop&blast_rank=1&RID=BAMM9CZF013) | 0.33 | 99 |
| *Corynebacterium falsenii* | [AF537594.1](http://www.ncbi.nlm.nih.gov/nucleotide/23954564?report=genbank&log$=nucltop&blast_rank=2&RID=BAMM9CZF013) | 0.33 | 99 |
| *Uncultured bacterium* | [GQ449199.1](http://www.ncbi.nlm.nih.gov/nucleotide/258548853?report=genbank&log$=nucltop&blast_rank=1&RID=BAMM9CZF013) | 0.33 | 99 |
| *Uncultured bacterium* | [GU605695.1](http://www.ncbi.nlm.nih.gov/nucleotide/290592288?report=genbank&log$=nucltop&blast_rank=1&RID=BAMM9CZF013) | 0.33 | 99 |
| *Uncultured bacterium* | [GU602554.1](http://www.ncbi.nlm.nih.gov/nucleotide/290589147?report=genbank&log$=nucltop&blast_rank=1&RID=BAMM9CZF013) | 0.33 | 99 |
| *Uncultured bacterium* | [FM253079.1](http://www.ncbi.nlm.nih.gov/nucleotide/238955415?report=genbank&log$=nucltop&blast_rank=1&RID=BAMM9CZF013) | 0.33 | 99 |
| *Psychrobacter marincola* | [AY292940.1](http://www.ncbi.nlm.nih.gov/nucleotide/34100977?report=genbank&log$=nucltop&blast_rank=1&RID=BAMM9CZF013) | 0.33 | 98 |
| *Uncultured bacterium* | [EF205694.1](http://www.ncbi.nlm.nih.gov/nucleotide/146285430?report=genbank&log$=nucltop&blast_rank=1&RID=BAMM9CZF013) | 0.33 | 100 |
| *Uncultured bacterium* | [HM318928.1](http://www.ncbi.nlm.nih.gov/nucleotide/297012523?report=genbank&log$=nucltop&blast_rank=1&RID=BAMM9CZF013) | 0.33 | 95 |
| *Histophilus somni* | [AB176902.1](http://www.ncbi.nlm.nih.gov/nucleotide/62122464?report=genbank&log$=nucltop&blast_rank=1&RID=BAMM9CZF013) | 0.33 | 99 |
| *Uncultured Porphyromonas spp.* | [HM754526.1](http://www.ncbi.nlm.nih.gov/nucleotide/304365992?report=genbank&log$=nucltop&blast_rank=1&RID=BAMM9CZF013) | 0.33 | 96 |
| *Uncultured bacterium* | [EU290137.1](http://www.ncbi.nlm.nih.gov/nucleotide/167595728?report=genbank&log$=nucltop&blast_rank=1&RID=BAMM9CZF013) | 0.33 | 94 |
| *Uncultured bacterium* | [GU612267.1](http://www.ncbi.nlm.nih.gov/nucleotide/290598861?report=genbank&log$=nucltop&blast_rank=1&RID=BAMM9CZF013) | 0.33 | 100 |
| *Uncultured bacterium* | [GU616751.1](http://www.ncbi.nlm.nih.gov/nucleotide/290603345?report=genbank&log$=nucltop&blast_rank=1&RID=BAMM9CZF013) | 0.33 | 100 |
| *Uncultured Clostridia bacterium* | [HM111527.1](http://www.ncbi.nlm.nih.gov/nucleotide/298396833?report=genbank&log$=nucltop&blast_rank=1&RID=BAMM9CZF013) | 0.33 | 87 |
| *Uncultured bacterium* | [GQ466866.1](http://www.ncbi.nlm.nih.gov/nucleotide/258549869?report=genbank&log$=nucltop&blast_rank=1&RID=BAMM9CZF013) | 0.33 | 99 |
