# Supplementary material for: Microbial Diversity of Bovine Mastitic Milk as Described by Pyrosequencing of Metagenomic 16s rDNA
Source: PLoS One. 2012 Oct 17;7(10):e47671. doi: 10.1371/journal.pone.0047671 (PMC3474744; doi:10.1371/journal.pone.0047671)
Supplement: Table S3 — Species level information (with GenBank Accession number, and identity match) for the predominant representative sequences in samples characterized as Escherichia coli mastitis. (DOCX) [file pone.0047671.s003.docx]

| Species | Accession No | Prevalence | Identity (%) |
| --- | --- | --- | --- |
| ***Escherichia coli*** | [CP003034.1](http://www.ncbi.nlm.nih.gov/nucleotide/349736152?report=genbank&log$=nucltop&blast_rank=1&RID=B54TYDFT016) | 10.12 | 100 |
| *Fusobacterium necrophorum subsp. funduliforme* | [AB525413.1](http://www.ncbi.nlm.nih.gov/nucleotide/261228522?report=genbank&log$=nucltop&blast_rank=5&RID=B54TYDFT016) | 8.21 | 99 |
| *Caulobacter leidyia* | [GQ891705.1](http://www.ncbi.nlm.nih.gov/nucleotide/260066246?report=genbank&log$=nucltop&blast_rank=6&RID=B54TYDFT016) | 7.67 | 99 |
| *Porphyromonas levii* | [AB547664.1](http://www.ncbi.nlm.nih.gov/nucleotide/302129302?report=genbank&log$=nucltop&blast_rank=1&RID=B54TYDFT016) | 3.87 | 100 |
| *Streptococcus uberis* | [HQ326695.1](http://www.ncbi.nlm.nih.gov/nucleotide/308390715?report=genbank&log$=nucltop&blast_rank=6&RID=B54TYDFT016) | 3.73 | 100 |
| *Uncultured bacterium* | [JF643239.1](http://www.ncbi.nlm.nih.gov/nucleotide/342078424?report=genbank&log$=nucltop&blast_rank=1&RID=B54TYDFT016) | 2.78 | 99 |
| *Uncultured bacterium* | [JF663845.1](http://www.ncbi.nlm.nih.gov/nucleotide/342099030?report=genbank&log$=nucltop&blast_rank=1&RID=B54TYDFT016) | 2.78 | 98 |
| *Uncultured bacterium* | [EF205686.1](http://www.ncbi.nlm.nih.gov/nucleotide/146285422?report=genbank&log$=nucltop&blast_rank=1&RID=B54TYDFT016) | 2.58 | 99 |
| *Staphylococcus equorum subsp. equorum* | [FR691468.1](http://www.ncbi.nlm.nih.gov/nucleotide/315002359?report=genbank&log$=nucltop&blast_rank=10&RID=B54TYDFT016) | 2.51 | 100 |
| *Uncultured Porphyromonas spp.* | [HM754526.1](http://www.ncbi.nlm.nih.gov/nucleotide/304365992?report=genbank&log$=nucltop&blast_rank=1&RID=B54TYDFT016) | 2.31 | 100 |
| *Uncultured bacterium* | [AM183009.1](http://www.ncbi.nlm.nih.gov/nucleotide/157690463?report=genbank&log$=nucltop&blast_rank=1&RID=B54TYDFT016) | 2.24 | 95 |
| *Uncultured Porphyromonas spp.* | [HM754526.1](http://www.ncbi.nlm.nih.gov/nucleotide/304365992?report=genbank&log$=nucltop&blast_rank=1&RID=B54TYDFT016) | 2.17 | 99 |
| *Ureaplasma diversum* | [NR_025878.1](http://www.ncbi.nlm.nih.gov/nucleotide/219846288?report=genbank&log$=nucltop&blast_rank=1&RID=B54TYDFT016) | 1.97 | 99 |
| *Uncultured bacterium* | [EU290118.1](http://www.ncbi.nlm.nih.gov/nucleotide/167595709?report=genbank&log$=nucltop&blast_rank=1&RID=B54TYDFT016) | 1.97 | 100 |
| *Ureaplasma diversum* | [NR_025878.1](http://www.ncbi.nlm.nih.gov/nucleotide/219846288?report=genbank&log$=nucltop&blast_rank=1&RID=B54TYDFT016) | 1.97 | 99 |
| *Uncultured bacterium* | [GU629689.1](http://www.ncbi.nlm.nih.gov/nucleotide/290616284?report=genbank&log$=nucltop&blast_rank=1&RID=B54TYDFT016) | 1.22 | 100 |
| *Bacteroides heparinolyticus* | [GQ422742.1](http://www.ncbi.nlm.nih.gov/nucleotide/257480655?report=genbank&log$=nucltop&blast_rank=3&RID=B54TYDFT016) | 1.15 | 100 |
| *Uncultured Prevotella spp.* | [GU905978.1](http://www.ncbi.nlm.nih.gov/nucleotide/294613820?report=genbank&log$=nucltop&blast_rank=2&RID=B54TYDFT016) | 1.15 | 99 |
| *Rumen bacterium* | [HM597702.1](http://www.ncbi.nlm.nih.gov/nucleotide/304569903?report=genbank&log$=nucltop&blast_rank=1&RID=B54TYDFT016) | 0.95 | 100 |
| *Uncultured Propionibacterium spp.* | [HQ891164.1](http://www.ncbi.nlm.nih.gov/nucleotide/336390587?report=genbank&log$=nucltop&blast_rank=3&RID=B54TYDFT016) | 0.95 | 100 |
| *Uncultured bacterium* | [HM318928.1](http://www.ncbi.nlm.nih.gov/nucleotide/297012523?report=genbank&log$=nucltop&blast_rank=1&RID=B54TYDFT016) | 0.88 | 95 |
| *Uncultured bacterium* | [FJ675143.1](http://www.ncbi.nlm.nih.gov/nucleotide/223679440?report=genbank&log$=nucltop&blast_rank=2&RID=B54TYDFT016) | 0.81 | 99 |
| *Paenibacillus spp.* | [GU733397.1](http://www.ncbi.nlm.nih.gov/nucleotide/322410177?report=genbank&log$=nucltop&blast_rank=1&RID=B54TYDFT016) | 0.81 | 99 |
| *Clostridium perfringens* | [AB627081.1](http://www.ncbi.nlm.nih.gov/nucleotide/332144243?report=genbank&log$=nucltop&blast_rank=1&RID=B54TYDFT016) | 0.68 | 100 |
| *Ochrobactrum pseudogrignonense* | [FJ859687.2](http://www.ncbi.nlm.nih.gov/nucleotide/272825711?report=genbank&log$=nucltop&blast_rank=1&RID=B54TYDFT016) | 0.68 | 99 |
| *Helcococcus ovis* | [AB542088.1](http://www.ncbi.nlm.nih.gov/nucleotide/284049428?report=genbank&log$=nucltop&blast_rank=10&RID=B54TYDFT016) | 0.54 | 100 |
| *Mycoplasma bovigenitalium* | [AY121109.1](http://www.ncbi.nlm.nih.gov/nucleotide/22122026?report=genbank&log$=nucltop&blast_rank=1&RID=B54TYDFT016) | 0.54 | 98 |
| *Histophilus somni* | [AB176910.1](http://www.ncbi.nlm.nih.gov/nucleotide/62122472?report=genbank&log$=nucltop&blast_rank=2&RID=B54TYDFT016) | 0.48 | 100 |
| *Peptostreptococcus anaerobius* | [AB640695.1](http://www.ncbi.nlm.nih.gov/nucleotide/336454741?report=genbank&log$=nucltop&blast_rank=1&RID=B54TYDFT016) | 0.48 | 97 |
| *Uncultured bacterium* | [EU458333.1](http://www.ncbi.nlm.nih.gov/nucleotide/169273808?report=genbank&log$=nucltop&blast_rank=1&RID=B54TYDFT016) | 0.48 | 99 |
| *Uncultured bacterium* | [FJ682454.1](http://www.ncbi.nlm.nih.gov/nucleotide/223695331?report=genbank&log$=nucltop&blast_rank=1&RID=B54TYDFT016) | 0.48 | 99 |
| *Uncultured Firmicutes* | [FR749723.1](http://www.ncbi.nlm.nih.gov/nucleotide/325973729?report=genbank&log$=nucltop&blast_rank=1&RID=B54TYDFT016) | 0.48 | 93 |
| *Uncultured Paludibacter spp.* | [EU794247.1](http://www.ncbi.nlm.nih.gov/nucleotide/192792298?report=genbank&log$=nucltop&blast_rank=5&RID=B54TYDFT016) | 0.41 | 100 |
| *Uncultured bacterium* | [FJ683872.1](http://www.ncbi.nlm.nih.gov/nucleotide/223696749?report=genbank&log$=nucltop&blast_rank=1&RID=B54TYDFT016) | 0.41 | 100 |
| *Prevotella spp.* | [FJ848548.1](http://www.ncbi.nlm.nih.gov/nucleotide/225733529?report=genbank&log$=nucltop&blast_rank=5&RID=B54TYDFT016) | 0.41 | 99 |
| *Uncultured bacterium* | [HM317008.1](http://www.ncbi.nlm.nih.gov/nucleotide/297010603?report=genbank&log$=nucltop&blast_rank=1&RID=B54TYDFT016) | 0.41 | 100 |
| *Uncultured bacterium* | [JN575965.1](http://www.ncbi.nlm.nih.gov/nucleotide/345847214?report=genbank&log$=nucltop&blast_rank=1&RID=B54TYDFT016) | 0.41 | 100 |
| *Streptococcus pasteurianus* | [JN581988.1](http://www.ncbi.nlm.nih.gov/nucleotide/346230216?report=genbank&log$=nucltop&blast_rank=1&RID=B54TYDFT016) | 0.41 | 99 |
| *Corynebacterium falsenii* | [AF537594.1](http://www.ncbi.nlm.nih.gov/nucleotide/23954564?report=genbank&log$=nucltop&blast_rank=2&RID=B54TYDFT016) | 0.34 | 99 |
| *Uncultured bacterium* | [AM183108.1](http://www.ncbi.nlm.nih.gov/nucleotide/157690562?report=genbank&log$=nucltop&blast_rank=1&RID=B54TYDFT016) | 0.34 | 92 |
| *Uncultured bacterium* | [GU629031.1](http://www.ncbi.nlm.nih.gov/nucleotide/290615626?report=genbank&log$=nucltop&blast_rank=1&RID=B54TYDFT016) | 0.34 | 100 |
| *Uncultured bacterium* | [JF905989.1](http://www.ncbi.nlm.nih.gov/nucleotide/348609497?report=genbank&log$=nucltop&blast_rank=1&RID=B54TYDFT016) | 0.34 | 99 |
