# Supplementary material for: Microbial Diversity of Bovine Mastitic Milk as Described by Pyrosequencing of Metagenomic 16s rDNA
Source: PLoS One. 2012 Oct 17;7(10):e47671. doi: 10.1371/journal.pone.0047671 (PMC3474744; doi:10.1371/journal.pone.0047671)
Supplement: Table S4 — Species level information (with GenBank Accession number, and identity match) for the predominant representative sequences in samples characterized as Klebsiella pneumoniae mastitis. (DOCX) [file pone.0047671.s004.docx]

| Species | Accession No | Prevalence | Identity (%) |
| --- | --- | --- | --- |
| ***Klebsiella pneumoniae*** | [AB675600.1](http://www.ncbi.nlm.nih.gov/nucleotide/353249747?report=genbank&log$=nucltop&blast_rank=1&RID=B5GDM67301S) | 13.20 | 100 |
| *Streptococcus uberis* | [HQ326695.1](http://www.ncbi.nlm.nih.gov/nucleotide/308390715?report=genbank&log$=nucltop&blast_rank=6&RID=B5GDM67301S) | 10.39 | 100 |
| *Caulobacter leidyia* | [GQ891705.1](http://www.ncbi.nlm.nih.gov/nucleotide/260066246?report=genbank&log$=nucltop&blast_rank=6&RID=B5GDM67301S) | 5.19 | 100 |
| *Fusobacterium necrophorum subsp. Funduliforme* | [AB525413.1](http://www.ncbi.nlm.nih.gov/nucleotide/261228522?report=genbank&log$=nucltop&blast_rank=5&RID=B5GDM67301S) | 4.45 | 100 |
| *Uncultured Bacteroides spp.* | [GU145757.2](http://www.ncbi.nlm.nih.gov/nucleotide/323696586?report=genbank&log$=nucltop&blast_rank=1&RID=B5GDM67301S) | 3.12 | 100 |
| *Uncultured bacterium* | [FJ682454.1](http://www.ncbi.nlm.nih.gov/nucleotide/223695331?report=genbank&log$=nucltop&blast_rank=1&RID=B5GDM67301S) | 3.12 | 99 |
| *Geobacillus pallidus* | [HM030740.1](http://www.ncbi.nlm.nih.gov/nucleotide/295853594?report=genbank&log$=nucltop&blast_rank=6&RID=B5GDM67301S) | 2.37 | 99 |
| *Uncultured bacterium* | [GU629689.1](http://www.ncbi.nlm.nih.gov/nucleotide/290616284?report=genbank&log$=nucltop&blast_rank=1&RID=B5GDM67301S) | 2.37 | 100 |
| *Uncultured bacterium* | [JF663845.1](http://www.ncbi.nlm.nih.gov/nucleotide/342099030?report=genbank&log$=nucltop&blast_rank=1&RID=B5GDM67301S) | 2.08 | 98 |
| *Uncultured Porphyromonas spp.* | [HM754526.1](http://www.ncbi.nlm.nih.gov/nucleotide/304365992?report=genbank&log$=nucltop&blast_rank=1&RID=B5GDM67301S) | 1.93 | 99 |
| *Porphyromonas levii* | [AB547664.1](http://www.ncbi.nlm.nih.gov/nucleotide/302129302?report=genbank&log$=nucltop&blast_rank=1&RID=B5GDM67301S) | 1.93 | 100 |
| *Paenibacillus borealis* | [HM563046.1](http://www.ncbi.nlm.nih.gov/nucleotide/302035379?report=genbank&log$=nucltop&blast_rank=1&RID=B5GDM67301S) | 1.78 | 99 |
| *Staphylococcus equorum subsp. equorum* | [FR691468.1](http://www.ncbi.nlm.nih.gov/nucleotide/315002359?report=genbank&log$=nucltop&blast_rank=10&RID=B5GDM67301S) | 1.63 | 100 |
| *Uncultured bacterium* | [JF643239.1](http://www.ncbi.nlm.nih.gov/nucleotide/342078424?report=genbank&log$=nucltop&blast_rank=1&RID=B5GDM67301S) | 1.48 | 100 |
| *Uncultured bacterium* | [FJ675143.1](http://www.ncbi.nlm.nih.gov/nucleotide/223679440?report=genbank&log$=nucltop&blast_rank=2&RID=B5GDM67301S) | 1.34 | 99 |
| *Prevotella spp.* | [FJ848548.1](http://www.ncbi.nlm.nih.gov/nucleotide/225733529?report=genbank&log$=nucltop&blast_rank=5&RID=B5GDM67301S) | 1.34 | 99 |
| *Uncultured bacterium* | [EU290118.1](http://www.ncbi.nlm.nih.gov/nucleotide/167595709?report=genbank&log$=nucltop&blast_rank=1&RID=B5GDM67301S) | 1.34 | 100 |
| *Bacteroides heparinolyticus* | [GQ422742.1](http://www.ncbi.nlm.nih.gov/nucleotide/257480655?report=genbank&log$=nucltop&blast_rank=3&RID=B5GDM67301S) | 1.04 | 100 |
| *Ureaplasma diversum* | [NR_025878.1](http://www.ncbi.nlm.nih.gov/nucleotide/219846288?report=genbank&log$=nucltop&blast_rank=1&RID=B5GDM67301S) | 0.89 | 99 |
| *Lactobacillus reuteri* | [JN092132.1](http://www.ncbi.nlm.nih.gov/nucleotide/341579618?report=genbank&log$=nucltop&blast_rank=1&RID=B5GDM67301S) | 0.89 | 99 |
| *Uncultured Clostridium spp.* | [HM235660.1](http://www.ncbi.nlm.nih.gov/nucleotide/301750633?report=genbank&log$=nucltop&blast_rank=1&RID=B5GDM67301S) | 0.89 | 98 |
| *Uncultured bacterium* | [HQ701538.1](http://www.ncbi.nlm.nih.gov/nucleotide/320097004?report=genbank&log$=nucltop&blast_rank=1&RID=B5GDM67301S) | 0.89 | 99 |
| *Uncultured Bacteroides spp.* | [EU289111.1](http://www.ncbi.nlm.nih.gov/nucleotide/162296268?report=genbank&log$=nucltop&blast_rank=9&RID=B5GDM67301S) | 0.89 | 100 |
| *Uncultured bacterium* | [JN575965.1](http://www.ncbi.nlm.nih.gov/nucleotide/345847214?report=genbank&log$=nucltop&blast_rank=1&RID=B5GDM67301S) | 0.74 | 100 |
| *Clostridium perfringens* | [FJ215342.1](http://www.ncbi.nlm.nih.gov/nucleotide/254841685?report=genbank&log$=nucltop&blast_rank=9&RID=B5GDM67301S) | 0.74 | 100 |
| *Clostridiales bacterium* | [HQ452852.1](http://www.ncbi.nlm.nih.gov/nucleotide/312064779?report=genbank&log$=nucltop&blast_rank=3&RID=B5GDM67301S) | 0.59 | 99 |
| *Bacteroides fragilis* | [FQ312004.1](http://www.ncbi.nlm.nih.gov/nucleotide/301161079?report=genbank&log$=nucltop&blast_rank=5&RID=B5GDM67301S) | 0.59 | 100 |
| *Fusobacterium necrophorum subsp. funduliforme* | [AB525413.1](http://www.ncbi.nlm.nih.gov/nucleotide/261228522?report=genbank&log$=nucltop&blast_rank=5&RID=B5GDM67301S) | 0.45 | 99 |
| *Uncultured bacterium* | [JF794810.1](http://www.ncbi.nlm.nih.gov/nucleotide/345296564?report=genbank&log$=nucltop&blast_rank=1&RID=B5GDM67301S) | 0.45 | 98 |
| *Uncultured Gram-positive bacterium* | [AB191022.1](http://www.ncbi.nlm.nih.gov/nucleotide/56541539?report=genbank&log$=nucltop&blast_rank=1&RID=B5GDM67301S) | 0.45 | 100 |
| *Uncultured bacterium* | [JN559616.1](http://www.ncbi.nlm.nih.gov/nucleotide/345294184?report=genbank&log$=nucltop&blast_rank=1&RID=B5GDM67301S) | 0.45 | 92 |
| *Uncultured bacterium* | [FJ683872.1](http://www.ncbi.nlm.nih.gov/nucleotide/223696749?report=genbank&log$=nucltop&blast_rank=1&RID=B5GDM67301S) | 0.45 | 100 |
| *Uncultured bacterium* | [JN575958.1](http://www.ncbi.nlm.nih.gov/nucleotide/345847207?report=genbank&log$=nucltop&blast_rank=1&RID=B5GDM67301S) | 0.45 | 99 |
| *Bacillus spp.* | [AM491453.2](http://www.ncbi.nlm.nih.gov/nucleotide/133740751?report=genbank&log$=nucltop&blast_rank=2&RID=B5GDM67301S) | 0.45 | 100 |
| *Uncultured bacterium* | [JF237709.1](http://www.ncbi.nlm.nih.gov/nucleotide/322223114?report=genbank&log$=nucltop&blast_rank=1&RID=B5GDM67301S) | 0.45 | 99 |
| *Uncultured bacterium* | [FJ683700.1](http://www.ncbi.nlm.nih.gov/nucleotide/223696577?report=genbank&log$=nucltop&blast_rank=1&RID=B5GDM67301S) | 0.45 | 99 |
| *Uncultured bacterium* | [EU460092.1](http://www.ncbi.nlm.nih.gov/nucleotide/169275567?report=genbank&log$=nucltop&blast_rank=1&RID=B5GDM67301S) | 0.45 | 97 |
| *Enterococcus faecalis* | [JN644614.1](http://www.ncbi.nlm.nih.gov/nucleotide/348161526?report=genbank&log$=nucltop&blast_rank=1&RID=B5GDM67301S) | 0.45 | 100 |
| *Uncultured Prevotella spp.* | [GU905979.1](http://www.ncbi.nlm.nih.gov/nucleotide/294613821?report=genbank&log$=nucltop&blast_rank=1&RID=B5GDM67301S) | 0.45 | 98 |
| *Uncultured bacterium* | [GU614672.1](http://www.ncbi.nlm.nih.gov/nucleotide/290601266?report=genbank&log$=nucltop&blast_rank=1&RID=B5GDM67301S) | 0.45 | 98 |
| *Uncultured Ruminococcus spp.* | [HM235655.1](http://www.ncbi.nlm.nih.gov/nucleotide/301750628?report=genbank&log$=nucltop&blast_rank=1&RID=B5GDM67301S) | 0.45 | 99 |
| *Uncultured bacterium* | [GQ449096.1](http://www.ncbi.nlm.nih.gov/nucleotide/258548750?report=genbank&log$=nucltop&blast_rank=1&RID=B5GDM67301S) | 0.45 | 100 |
| *Streptococcus dysgalactiae subsp. dysgalactiae* | [EF151154.1](http://www.ncbi.nlm.nih.gov/nucleotide/120568239?report=genbank&log$=nucltop&blast_rank=1&RID=B5GDM67301S) | 0.45 | 99 |
