# Supplementary material for: Microbial Diversity of Bovine Mastitic Milk as Described by Pyrosequencing of Metagenomic 16s rDNA
Source: PLoS One. 2012 Oct 17;7(10):e47671. doi: 10.1371/journal.pone.0047671 (PMC3474744; doi:10.1371/journal.pone.0047671)
Supplement: Table S5 — Species level information (with GenBank Accession number, and identity match) for the predominant representative sequences in samples characterized as Staphylococcus aureus mastitis. (DOCX) [file pone.0047671.s005.docx]

| Species | Accession No | Prevalence | Identity (%) |
| --- | --- | --- | --- |
| *Caulobacter leidyia* | [GQ891705.1](http://www.ncbi.nlm.nih.gov/nucleotide/260066246?report=genbank&log$=nucltop&blast_rank=6&RID=BG1NCPM101S) | 11.12 | 99 |
| *Fusobacterium necrophorum subsp. funduliforme* | [AB525413.1](http://www.ncbi.nlm.nih.gov/nucleotide/261228522?report=genbank&log$=nucltop&blast_rank=5&RID=BG1NCPM101S) | 6.44 | 100 |
| ***Staphylococcus aureus subsp. aureus*** | [FR821779.1](http://www.ncbi.nlm.nih.gov/nucleotide/344176319?report=genbank&log$=nucltop&blast_rank=1&RID=BG1NCPM101S) | 6.39 | 100 |
| *Uncultured bacterium* | [JF643239.1](http://www.ncbi.nlm.nih.gov/nucleotide/342078424?report=genbank&log$=nucltop&blast_rank=1&RID=BG1NCPM101S) | 5.96 | 100 |
| *Uncultured bacterium* | [FJ657725.1](http://www.ncbi.nlm.nih.gov/nucleotide/223954580?report=genbank&log$=nucltop&blast_rank=1&RID=BG1NCPM101S) | 4.19 | 100 |
| *Porphyromonas levii* | [FJ822532.1](http://www.ncbi.nlm.nih.gov/nucleotide/225795241?report=genbank&log$=nucltop&blast_rank=5&RID=BG1NCPM101S) | 3.28 | 100 |
| *Uncultured bacterium* | [JF663845.1](http://www.ncbi.nlm.nih.gov/nucleotide/342099030?report=genbank&log$=nucltop&blast_rank=1&RID=BG1NCPM101S) | 3.11 | 98 |
| *Uncultured Prevotella spp.* | [GU905978.1](http://www.ncbi.nlm.nih.gov/nucleotide/294613820?report=genbank&log$=nucltop&blast_rank=2&RID=BG1NCPM101S) | 2.15 | 99 |
| *Propionibacterium acnes* | [CP003084.1](http://www.ncbi.nlm.nih.gov/nucleotide/353454017?report=genbank&log$=nucltop&blast_rank=1&RID=BG1NCPM101S) | 2.15 | 100 |
| *Uncultured Porphyromonas spp.* | [HM754526.1](http://www.ncbi.nlm.nih.gov/nucleotide/304365992?report=genbank&log$=nucltop&blast_rank=1&RID=BG1NCPM101S) | 2.15 | 98 |
| *Uncultured Porphyromonas spp.* | [HM754526.1](http://www.ncbi.nlm.nih.gov/nucleotide/304365992?report=genbank&log$=nucltop&blast_rank=1&RID=BG1NCPM101S) | 1.99 | 100 |
| *Uncultured bacterium* | [AM183009.1](http://www.ncbi.nlm.nih.gov/nucleotide/157690463?report=genbank&log$=nucltop&blast_rank=1&RID=BG1NCPM101S) | 1.88 | 95 |
| *Streptococcus uberis* | [HQ326695.1](http://www.ncbi.nlm.nih.gov/nucleotide/308390715?report=genbank&log$=nucltop&blast_rank=6&RID=BG1NCPM101S) | 1.61 | 100 |
| *Swine manure pit bacterium* | [AF445295.1](http://www.ncbi.nlm.nih.gov/nucleotide/17940546?report=genbank&log$=nucltop&blast_rank=1&RID=BG1NCPM101S) | 1.40 | 99 |
| *Staphylococcus equorum subsp. linens* | [NR_041926.1](http://www.ncbi.nlm.nih.gov/nucleotide/343198492?report=genbank&log$=nucltop&blast_rank=10&RID=BG1NCPM101S) | 1.40 | 100 |
| *Bacteroides heparinolyticus* | [GQ422742.1](http://www.ncbi.nlm.nih.gov/nucleotide/257480655?report=genbank&log$=nucltop&blast_rank=3&RID=BG1NCPM101S) | 1.34 | 100 |
| *Uncultured Bacteroides spp.* | [EU289111.1](http://www.ncbi.nlm.nih.gov/nucleotide/162296268?report=genbank&log$=nucltop&blast_rank=9&RID=BG1NCPM101S) | 1.29 | 100 |
| *Uncultured Clostridiales bacterium* | [HM080230.1](http://www.ncbi.nlm.nih.gov/nucleotide/297346684?report=genbank&log$=nucltop&blast_rank=4&RID=BG1NCPM101S) | 1.24 | 94 |
| *Paenibacillus borealis* | [HM563046.1](http://www.ncbi.nlm.nih.gov/nucleotide/302035379?report=genbank&log$=nucltop&blast_rank=1&RID=BG1NCPM101S) | 1.18 | 98 |
| *Ureaplasma diversum* | [NR_025878.1](http://www.ncbi.nlm.nih.gov/nucleotide/219846288?report=genbank&log$=nucltop&blast_rank=1&RID=BG1NCPM101S) | 1.13 | 99 |
| *Prevotella spp.* | [FJ848548.1](http://www.ncbi.nlm.nih.gov/nucleotide/225733529?report=genbank&log$=nucltop&blast_rank=5&RID=BG1NCPM101S) | 0.97 | 100 |
| *Uncultured bacterium* | [HM317008.1](http://www.ncbi.nlm.nih.gov/nucleotide/297010603?report=genbank&log$=nucltop&blast_rank=1&RID=BG1NCPM101S) | 0.86 | 100 |
| *Uncultured bacterium* | [EU290118.1](http://www.ncbi.nlm.nih.gov/nucleotide/167595709?report=genbank&log$=nucltop&blast_rank=1&RID=BG1NCPM101S) | 0.86 | 100 |
| *Helcococcus ovis* | [AB542088.1](http://www.ncbi.nlm.nih.gov/nucleotide/284049428?report=genbank&log$=nucltop&blast_rank=10&RID=BG1NCPM101S) | 0.70 | 100 |
| *Mycoplasma bovigenitalium* | [AY121109.1](http://www.ncbi.nlm.nih.gov/nucleotide/22122026?report=genbank&log$=nucltop&blast_rank=1&RID=BG1NCPM101S) | 0.64 | 99 |
| *Uncultured bacterium* | [EU290005.1](http://www.ncbi.nlm.nih.gov/nucleotide/167595596?report=genbank&log$=nucltop&blast_rank=1&RID=BG1NCPM101S) | 0.59 | 100 |
| *Histophilus somni* | [AB176913.1](http://www.ncbi.nlm.nih.gov/nucleotide/62122475?report=genbank&log$=nucltop&blast_rank=1&RID=BG1NCPM101S) | 0.54 | 100 |
| *Halomonas desiderata* | [AB362300.1](http://www.ncbi.nlm.nih.gov/nucleotide/171703139?report=genbank&log$=nucltop&blast_rank=3&RID=BG1NCPM101S) | 0.48 | 100 |
| *Corynebacterium falsenii* | [AF537594.1](http://www.ncbi.nlm.nih.gov/nucleotide/23954564?report=genbank&log$=nucltop&blast_rank=2&RID=BG1NCPM101S) | 0.43 | 100 |
| *Ochrobactrum pseudogrignonense* | [FJ859687.2](http://www.ncbi.nlm.nih.gov/nucleotide/272825711?report=genbank&log$=nucltop&blast_rank=1&RID=BG1NCPM101S) | 0.43 | 100 |
| *Trueperella pyogenes* | [JN578112.1](http://www.ncbi.nlm.nih.gov/nucleotide/345847767?report=genbank&log$=nucltop&blast_rank=1&RID=BG1NCPM101S) | 0.43 | 99 |
| *Uncultured bacterium* | [JN230113.1](http://www.ncbi.nlm.nih.gov/nucleotide/345122147?report=genbank&log$=nucltop&blast_rank=1&RID=BG1NCPM101S) | 0.38 | 99 |
| *Bacillus psychrodurans* | [GU385871.1](http://www.ncbi.nlm.nih.gov/nucleotide/289186790?report=genbank&log$=nucltop&blast_rank=3&RID=BG1NCPM101S) | 0.38 | 99 |
| *Escherichia coli* | [JN180967.1](http://www.ncbi.nlm.nih.gov/nucleotide/341618717?report=genbank&log$=nucltop&blast_rank=5&RID=BG1NCPM101S) | 0.38 | 100 |
| *Uncultured bacterium* | [GU614651.1](http://www.ncbi.nlm.nih.gov/nucleotide/290601245?report=genbank&log$=nucltop&blast_rank=1&RID=BG1NCPM101S) | 0.38 | 99 |
| *Uncultured bacterium* | [GU617155.1](http://www.ncbi.nlm.nih.gov/nucleotide/290603749?report=genbank&log$=nucltop&blast_rank=1&RID=BG1NCPM101S) | 0.38 | 100 |
| *Uncultured bacterium* | [FJ684368.1](http://www.ncbi.nlm.nih.gov/nucleotide/223688665?report=genbank&log$=nucltop&blast_rank=1&RID=BG1NCPM101S) | 0.38 | 99 |
| *Uncultured bacterium* | [EU458333.1](http://www.ncbi.nlm.nih.gov/nucleotide/169273808?report=genbank&log$=nucltop&blast_rank=1&RID=BG1NCPM101S) | 0.32 | 98 |
| *Uncultured bacterium* | [GU600588.1](http://www.ncbi.nlm.nih.gov/nucleotide/290587181?report=genbank&log$=nucltop&blast_rank=1&RID=BG1NCPM101S) | 0.32 | 98 |
| *Streptococcus dysgalactiae subsp. dysgalactiae* | [EF151154.1](http://www.ncbi.nlm.nih.gov/nucleotide/120568239?report=genbank&log$=nucltop&blast_rank=1&RID=BG1NCPM101S) | 0.32 | 100 |
