# Supplementary material for: Microbial Diversity of Bovine Mastitic Milk as Described by Pyrosequencing of Metagenomic 16s rDNA
Source: PLoS One. 2012 Oct 17;7(10):e47671. doi: 10.1371/journal.pone.0047671 (PMC3474744; doi:10.1371/journal.pone.0047671)
Supplement: Table S6 — Species level information (with GenBank Accession number, and identity match) for the predominant representative sequences in samples characterized as Staphylococcus spp. Mastitis. (DOCX) [file pone.0047671.s006.docx]

| Species | Accession No | Prevalence | Identity (%) |
| --- | --- | --- | --- |
| *Caulobacter leidyia (OTU1)* | [GQ891705.1](http://www.ncbi.nlm.nih.gov/nucleotide/260066246?report=genbank&log$=nucltop&blast_rank=6&RID=BNRBCK2801S) | 18.91 | 98 |
| *Fusobacterium necrophorum subsp. Funduliforme (OTU2)* | [AB525413.1](http://www.ncbi.nlm.nih.gov/nucleotide/261228522?report=genbank&log$=nucltop&blast_rank=5&RID=BNRBCK2801S) | 5.14 | 100 |
| *Uncultured bacterium (OTU3)* | [JF643239.1](http://www.ncbi.nlm.nih.gov/nucleotide/342078424?report=genbank&log$=nucltop&blast_rank=1&RID=BNRBCK2801S) | 4.82 | 100 |
| *Geobacillus pallidus (OTU4)* | [HM030740.1](http://www.ncbi.nlm.nih.gov/nucleotide/295853594?report=genbank&log$=nucltop&blast_rank=4&RID=BNRBCK2801S) | 3.86 | 99 |
| *Uncultured bacterium (OTU5)* | [JF663845.1](http://www.ncbi.nlm.nih.gov/nucleotide/342099030?report=genbank&log$=nucltop&blast_rank=1&RID=BNRBCK2801S) | 3.60 | 98 |
| *Porphyromonas levii (OTU6)* | [AB547664.1](http://www.ncbi.nlm.nih.gov/nucleotide/302129302?report=genbank&log$=nucltop&blast_rank=1&RID=BNRBCK2801S) | 2.25 | 100 |
| *Uncultured bacterium (OTU7)* | [FJ675143.1](http://www.ncbi.nlm.nih.gov/nucleotide/223679440?report=genbank&log$=nucltop&blast_rank=2&RID=BNRBCK2801S) | 1.93 | 99 |
| *Uncultured Prevotella spp. (OTU8)* | [GU905979.1](http://www.ncbi.nlm.nih.gov/nucleotide/294613821?report=genbank&log$=nucltop&blast_rank=1&RID=BNRBCK2801S) | 1.80 | 98 |
| ***Staphylococcus equorum subsp. linens (OTU9)*** | [NR_041926.1](http://www.ncbi.nlm.nih.gov/nucleotide/343198492?report=genbank&log$=nucltop&blast_rank=10&RID=BNRBCK2801S) | 1.80 | 100 |
| *Propionibacterium acnes (OTU10)* | [CP003084.1](http://www.ncbi.nlm.nih.gov/nucleotide/353454017?report=genbank&log$=nucltop&blast_rank=1&RID=BNRBCK2801S) | 1.74 | 100 |
| *Uncultured Porphyromonas spp.* | [HM754526.1](http://www.ncbi.nlm.nih.gov/nucleotide/304365992?report=genbank&log$=nucltop&blast_rank=1&RID=BNRBCK2801S) | 1.41 | 100 |
| *Bacteroides heparinolyticus* | [GQ422742.1](http://www.ncbi.nlm.nih.gov/nucleotide/257480655?report=genbank&log$=nucltop&blast_rank=3&RID=BNRBCK2801S) | 1.41 | 100 |
| *Paenibacillus borealis* | [HM563046.1](http://www.ncbi.nlm.nih.gov/nucleotide/302035379?report=genbank&log$=nucltop&blast_rank=1&RID=BNRBCK2801S) | 1.35 | 98 |
| *Rumen bacterium* | [HM597702.1](http://www.ncbi.nlm.nih.gov/nucleotide/304569903?report=genbank&log$=nucltop&blast_rank=1&RID=BNRBCK2801S) | 1.16 | 100 |
| *Uncultured Porphyromonas spp.* | [HM754526.1](http://www.ncbi.nlm.nih.gov/nucleotide/304365992?report=genbank&log$=nucltop&blast_rank=1&RID=BNRBCK2801S) | 1.09 | 98 |
| *Ureaplasma diversum* | [NR_025878.1](http://www.ncbi.nlm.nih.gov/nucleotide/219846288?report=genbank&log$=nucltop&blast_rank=1&RID=BNRBCK2801S) | 1.03 | 98 |
| *Uncultured bacterium* | [AM183009.1](http://www.ncbi.nlm.nih.gov/nucleotide/157690463?report=genbank&log$=nucltop&blast_rank=1&RID=BNRBCK2801S) | 0.90 | 95 |
| *Uncultured bacterium* | [EU845721.1](http://www.ncbi.nlm.nih.gov/nucleotide/194140284?report=genbank&log$=nucltop&blast_rank=1&RID=BNRBCK2801S) | 0.84 | 99 |
| *Ochrobactrum pseudogrignonense* | [FJ859687.2](http://www.ncbi.nlm.nih.gov/nucleotide/272825711?report=genbank&log$=nucltop&blast_rank=1&RID=BNRBCK2801S) | 0.84 | 99 |
| *Prevotella spp.* | [FJ848548.1](http://www.ncbi.nlm.nih.gov/nucleotide/225733529?report=genbank&log$=nucltop&blast_rank=5&RID=BNRBCK2801S) | 0.84 | 100 |
| *Uncultured bacterium* | [HM317008.1](http://www.ncbi.nlm.nih.gov/nucleotide/297010603?report=genbank&log$=nucltop&blast_rank=1&RID=BNRBCK2801S) | 0.71 | 100 |
| *Uncultured bacterium* | [HM318928.1](http://www.ncbi.nlm.nih.gov/nucleotide/297012523?report=genbank&log$=nucltop&blast_rank=1&RID=BNRBCK2801S) | 0.64 | 95 |
| *Streptococcus uberis* | [HQ326695.1](http://www.ncbi.nlm.nih.gov/nucleotide/308390715?report=genbank&log$=nucltop&blast_rank=6&RID=BNRBCK2801S) | 0.58 | 100 |
| *Uncultured bacterium* | [EU290118.1](http://www.ncbi.nlm.nih.gov/nucleotide/167595709?report=genbank&log$=nucltop&blast_rank=1&RID=BNRBCK2801S) | 0.58 | 100 |
| *Uncultured bacterium* | [FN436078.1](http://www.ncbi.nlm.nih.gov/nucleotide/255976702?report=genbank&log$=nucltop&blast_rank=1&RID=BNRBCK2801S) | 0.51 | 96 |
| *Escherichia coli* | [CP003034.1](http://www.ncbi.nlm.nih.gov/nucleotide/349736152?report=genbank&log$=nucltop&blast_rank=4&RID=BNRBCK2801S) | 0.51 | 100 |
| *Uncultured bacterium* | [GU616649.1](http://www.ncbi.nlm.nih.gov/nucleotide/290603243?report=genbank&log$=nucltop&blast_rank=1&RID=BNRBCK2801S) | 0.51 | 99 |
| *Uncultured bacterium* | [GU608491.1](http://www.ncbi.nlm.nih.gov/nucleotide/290595084?report=genbank&log$=nucltop&blast_rank=1&RID=BNRBCK2801S) | 0.51 | 99 |
| *Uncultured bacterium* | [GU602110.1](http://www.ncbi.nlm.nih.gov/nucleotide/290588703?report=genbank&log$=nucltop&blast_rank=1&RID=BNRBCK2801S) | 0.45 | 98 |
| *Uncultured bacterium* | [EU464157.1](http://www.ncbi.nlm.nih.gov/nucleotide/169279632?report=genbank&log$=nucltop&blast_rank=1&RID=BNRBCK2801S) | 0.45 | 99 |
| *Histophilus somni* | [AB176913.1](http://www.ncbi.nlm.nih.gov/nucleotide/62122475?report=genbank&log$=nucltop&blast_rank=1&RID=BNRBCK2801S) | 0.45 | 100 |
| *Helcococcus ovis* | [AB542088.1](http://www.ncbi.nlm.nih.gov/nucleotide/284049428?report=genbank&log$=nucltop&blast_rank=10&RID=BNRBCK2801S) | 0.45 | 99 |
| *Mycoplasma bovigenitalium* | [AY121109.1](http://www.ncbi.nlm.nih.gov/nucleotide/22122026?report=genbank&log$=nucltop&blast_rank=1&RID=BNRBCK2801S) | 0.39 | 100 |
| *Uncultured Bacteroidetes* | [FM252970.1](http://www.ncbi.nlm.nih.gov/nucleotide/238955306?report=genbank&log$=nucltop&blast_rank=2&RID=BNRBCK2801S) | 0.39 | 100 |
| *Uncultured bacterium* | [JF810468.1](http://www.ncbi.nlm.nih.gov/nucleotide/334690322?report=genbank&log$=nucltop&blast_rank=1&RID=BNRBCK2801S) | 0.39 | 100 |
| *Corynebacterium falsenii* | [AF537594.1](http://www.ncbi.nlm.nih.gov/nucleotide/23954564?report=genbank&log$=nucltop&blast_rank=2&RID=BNRBCK2801S) | 0.32 | 100 |
| *Paenibacillus caespitis* | [AM745263.1](http://www.ncbi.nlm.nih.gov/nucleotide/150246971?report=genbank&log$=nucltop&blast_rank=1&RID=BNRBCK2801S) | 0.32 | 99 |
| *Uncultured bacterium* | [GQ449221.1](http://www.ncbi.nlm.nih.gov/nucleotide/258548875?report=genbank&log$=nucltop&blast_rank=1&RID=BNRBCK2801S) | 0.32 | 98 |
| *Pseudomonas saccharophila* | [AF368755.1](http://www.ncbi.nlm.nih.gov/nucleotide/14091482?report=genbank&log$=nucltop&blast_rank=9&RID=BNRBCK2801S) | 0.32 | 100 |
| *Enterococcus spp.* | [AF445307.2](http://www.ncbi.nlm.nih.gov/nucleotide/339249878?report=genbank&log$=nucltop&blast_rank=1&RID=BNRBCK2801S) | 0.32 | 99 |
| *Uncultured bacterium* | [FN994146.1](http://www.ncbi.nlm.nih.gov/nucleotide/304656162?report=genbank&log$=nucltop&blast_rank=1&RID=BNRBCK2801S) | 0.32 | 93 |
| *Psychrobacter aquaticus* | [NR_042206.1](http://www.ncbi.nlm.nih.gov/nucleotide/343201480?report=genbank&log$=nucltop&blast_rank=9&RID=BNRBCK2801S) | 0.32 | 100 |
| *Uncultured bacterium* | [EU458333.1](http://www.ncbi.nlm.nih.gov/nucleotide/169273808?report=genbank&log$=nucltop&blast_rank=1&RID=BNRBCK2801S) | 0.32 | 99 |
| *Uncultured Bacteroidales* | [EU794077.1](http://www.ncbi.nlm.nih.gov/nucleotide/192792128?report=genbank&log$=nucltop&blast_rank=10&RID=BNRBCK2801S) | 0.32 | 98 |
| *Uncultured bacterium* | [GU608708.1](http://www.ncbi.nlm.nih.gov/nucleotide/290595301?report=genbank&log$=nucltop&blast_rank=1&RID=BNRBCK2801S) | 0.32 | 99 |
| *Arthrobacter spp.* | [AM260537.1](http://www.ncbi.nlm.nih.gov/nucleotide/107593744?report=genbank&log$=nucltop&blast_rank=7&RID=BNRBCK2801S) | 0.32 | 100 |
| *Uncultured bacterium* | [AM982606.1](http://www.ncbi.nlm.nih.gov/nucleotide/186929227?report=genbank&log$=nucltop&blast_rank=1&RID=BNRBCK2801S) | 0.32 | 98 |
| *Uncultured Lactococcus spp.* | [GQ464389.1](http://www.ncbi.nlm.nih.gov/nucleotide/260595160?report=genbank&log$=nucltop&blast_rank=1&RID=BNRBCK2801S) | 0.32 | 98 |
