# Supplementary material for: Microbial Diversity of Bovine Mastitic Milk as Described by Pyrosequencing of Metagenomic 16s rDNA
Source: PLoS One. 2012 Oct 17;7(10):e47671. doi: 10.1371/journal.pone.0047671 (PMC3474744; doi:10.1371/journal.pone.0047671)
Supplement: Table S7 — Species level information (with GenBank Accession number, and identity match) for the predominant representative sequences in samples characterized as Streptococcus dysgalactiae mastitis. (DOCX) [file pone.0047671.s007.docx]

| Species | Accession No | Prevalence | Identity (%) |
| --- | --- | --- | --- |
| *Streptococcus uberis* | [HQ326695.1](http://www.ncbi.nlm.nih.gov/nucleotide/308390715?report=genbank&log$=nucltop&blast_rank=6&RID=BNVWF13001N) | 6.97 | 100 |
| ***Streptococcus dysgalactiae subsp. dysgalactiae*** | [EF151154.1](http://www.ncbi.nlm.nih.gov/nucleotide/120568239?report=genbank&log$=nucltop&blast_rank=1&RID=BNVWF13001N) | 6.25 | 100 |
| *Uncultured bacterium* | [JF643239.1](http://www.ncbi.nlm.nih.gov/nucleotide/342078424?report=genbank&log$=nucltop&blast_rank=1&RID=BNVWF13001N) | 5.46 | 99 |
| *Porphyromonas levii* | [AB547664.1](http://www.ncbi.nlm.nih.gov/nucleotide/302129302?report=genbank&log$=nucltop&blast_rank=1&RID=BNVWF13001N) | 5.07 | 99 |
| *Uncultured bacterium* | [FJ657725.1](http://www.ncbi.nlm.nih.gov/nucleotide/223954580?report=genbank&log$=nucltop&blast_rank=1&RID=BNVWF13001N) | 4.35 | 100 |
| *Uncultured Fusobacteria* | [EF704825.1](http://www.ncbi.nlm.nih.gov/nucleotide/154197591?report=genbank&log$=nucltop&blast_rank=1&RID=BNVWF13001N) | 4.35 | 100 |
| *Caulobacter leidyia* | [GQ891705.1](http://www.ncbi.nlm.nih.gov/nucleotide/260066246?report=genbank&log$=nucltop&blast_rank=6&RID=BNVWF13001N) | 4.12 | 100 |
| *Uncultured Porphyromonas spp.* | [HM754526.1](http://www.ncbi.nlm.nih.gov/nucleotide/304365992?report=genbank&log$=nucltop&blast_rank=1&RID=BNVWF13001N) | 4.04 | 100 |
| *Uncultured bacterium* | [JF663845.1](http://www.ncbi.nlm.nih.gov/nucleotide/342099030?report=genbank&log$=nucltop&blast_rank=1&RID=BNVWF13001N) | 3.01 | 98 |
| *Propionibacterium acnes* | [CP003084.1](http://www.ncbi.nlm.nih.gov/nucleotide/353454017?report=genbank&log$=nucltop&blast_rank=1&RID=BNVWF13001N) | 2.69 | 100 |
| *Uncultured bacterium* | [AM183009.1](http://www.ncbi.nlm.nih.gov/nucleotide/157690463?report=genbank&log$=nucltop&blast_rank=1&RID=BNVWF13001N) | 2.45 | 95 |
| *Uncultured Bacteroides spp.* | [EU289070.1](http://www.ncbi.nlm.nih.gov/nucleotide/162296227?report=genbank&log$=nucltop&blast_rank=10&RID=BNVWF13001N) | 2.06 | 99 |
| *Uncultured Porphyromonas spp.* | [HM754526.1](http://www.ncbi.nlm.nih.gov/nucleotide/304365992?report=genbank&log$=nucltop&blast_rank=1&RID=BNVWF13001N) | 1.98 | 99 |
| *Uncultured Prevotella spp.* | [GU905979.1](http://www.ncbi.nlm.nih.gov/nucleotide/294613821?report=genbank&log$=nucltop&blast_rank=1&RID=BNVWF13001N) | 1.82 | 98 |
| *Uncultured bacterium* | [HM318928.1](http://www.ncbi.nlm.nih.gov/nucleotide/297012523?report=genbank&log$=nucltop&blast_rank=1&RID=BNVWF13001N) | 1.74 | 94 |
| *Uncultured bacterium* | [JF810468.1](http://www.ncbi.nlm.nih.gov/nucleotide/334690322?report=genbank&log$=nucltop&blast_rank=1&RID=BNVWF13001N) | 0.71 | 100 |
| *Veillonella dispar* | [GU404460.1](http://www.ncbi.nlm.nih.gov/nucleotide/285168464?report=genbank&log$=nucltop&blast_rank=10&RID=BNVWF13001N) | 0.63 | 89 |
| *Histophilus somni* | [AB176910.1](http://www.ncbi.nlm.nih.gov/nucleotide/62122472?report=genbank&log$=nucltop&blast_rank=2&RID=BNVWF13001N) | 0.63 | 100 |
| *Ochrobactrum pseudogrignonense* | [FJ859687.2](http://www.ncbi.nlm.nih.gov/nucleotide/272825711?report=genbank&log$=nucltop&blast_rank=1&RID=BNVWF13001N) | 0.63 | 99 |
| *Paenibacillus borealis* | [HM563046.1](http://www.ncbi.nlm.nih.gov/nucleotide/302035379?report=genbank&log$=nucltop&blast_rank=1&RID=BNVWF13001N) | 0.55 | 99 |
| *Helcococcus ovis* | [AB542088.1](http://www.ncbi.nlm.nih.gov/nucleotide/284049428?report=genbank&log$=nucltop&blast_rank=10&RID=BNVWF13001N) | 0.55 | 100 |
| *Uncultured bacterium* | [JN575965.1](http://www.ncbi.nlm.nih.gov/nucleotide/345847214?report=genbank&log$=nucltop&blast_rank=1&RID=BNVWF13001N) | 0.55 | 100 |
| *Bacteroides fragilis* | [FQ312004.1](http://www.ncbi.nlm.nih.gov/nucleotide/301161079?report=genbank&log$=nucltop&blast_rank=5&RID=BNVWF13001N) | 0.55 | 100 |
| *Halomonas spp.* | [AJ302088.1](http://www.ncbi.nlm.nih.gov/nucleotide/12697323?report=genbank&log$=nucltop&blast_rank=1&RID=BNVWF13001N) | 0.48 | 99 |
| *Uncultured bacterium* | [JN575958.1](http://www.ncbi.nlm.nih.gov/nucleotide/345847207?report=genbank&log$=nucltop&blast_rank=1&RID=BNVWF13001N) | 0.48 | 100 |
| *Uncultured bacterium* | [EU460092.1](http://www.ncbi.nlm.nih.gov/nucleotide/169275567?report=genbank&log$=nucltop&blast_rank=1&RID=BNVWF13001N) | 0.48 | 97 |
| *Staphylococcus equorum subsp. linens* | [NR_041926.1](http://www.ncbi.nlm.nih.gov/nucleotide/343198492?report=genbank&log$=nucltop&blast_rank=10&RID=BNVWF13001N) | 0.40 | 99 |
| *Uncultured Gram-positive bacterium* | [AB191022.1](http://www.ncbi.nlm.nih.gov/nucleotide/56541539?report=genbank&log$=nucltop&blast_rank=1&RID=BNVWF13001N) | 0.40 | 98 |
| *Trueperella pyogenes* | [JN578141.1](http://www.ncbi.nlm.nih.gov/nucleotide/345847796?report=genbank&log$=nucltop&blast_rank=1&RID=BNVWF13001N) | 0.40 | 100 |
| *Uncultured Corynebacterium spp.* | [JN082697.1](http://www.ncbi.nlm.nih.gov/nucleotide/341831959?report=genbank&log$=nucltop&blast_rank=2&RID=BNVWF13001N) | 0.32 | 100 |
| *Clostridium perfringens str.* | [BA000016.3](http://www.ncbi.nlm.nih.gov/nucleotide/47118322?report=genbank&log$=nucltop&blast_rank=6&RID=BNVWF13001N) | 0.32 | 100 |
| *Uncultured Ruminococcus spp.* | [HM235655.1](http://www.ncbi.nlm.nih.gov/nucleotide/301750628?report=genbank&log$=nucltop&blast_rank=1&RID=BNVWF13001N) | 0.32 | 100 |
| *Psychrobacter marincola* | [AY292940.1](http://www.ncbi.nlm.nih.gov/nucleotide/34100977?report=genbank&log$=nucltop&blast_rank=1&RID=BNVWF13001N) | 0.32 | 98 |
| *Mycoplasma bovigenitalium* | [AY121109.1](http://www.ncbi.nlm.nih.gov/nucleotide/22122026?report=genbank&log$=nucltop&blast_rank=1&RID=BNVWF13001N) | 0.32 | 100 |
| *Uncultured bacterium* | [GU608534.1](http://www.ncbi.nlm.nih.gov/nucleotide/290595127?report=genbank&log$=nucltop&blast_rank=1&RID=BNVWF13001N) | 0.32 | 99 |
| *Bacillus spp.* | [FR774960.1](http://www.ncbi.nlm.nih.gov/nucleotide/333755779?report=genbank&log$=nucltop&blast_rank=1&RID=BNVWF13001N) | 0.32 | 100 |
