# Supplementary material for: Microbial Diversity of Bovine Mastitic Milk as Described by Pyrosequencing of Metagenomic 16s rDNA
Source: PLoS One. 2012 Oct 17;7(10):e47671. doi: 10.1371/journal.pone.0047671 (PMC3474744; doi:10.1371/journal.pone.0047671)
Supplement: Table S8 — Species level information (with GenBank Accession number, and identity match) for the predominant representative sequences in samples characterized as Streptococcus spp. Mastitis. (DOCX) [file pone.0047671.s008.docx]

**Table 8.** Species level information (with GenBank Accession number, and identity match) for the predominant representative sequences in samples characterized as *Streptococcus* spp*.* mastitis

| Species | Accession No | Prevalence | Identity (%) |
| --- | --- | --- | --- |
| ***Streptococcus macedonicus*** | [AF459431.1](http://www.ncbi.nlm.nih.gov/nucleotide/23477251?report=genbank&log$=nucltop&blast_rank=4&RID=BGS136YC01S) | 18.54 | 100 |
| *Uncultured Porphyromonas spp.* | [HM754526.1](http://www.ncbi.nlm.nih.gov/nucleotide/304365992?report=genbank&log$=nucltop&blast_rank=1&RID=BGS136YC01S) | 11.92 | 99 |
| *Uncultured Fusobacteria* | [EF704825.1](http://www.ncbi.nlm.nih.gov/nucleotide/154197591?report=genbank&log$=nucltop&blast_rank=1&RID=BGS136YC01S) | 9.27 | 100 |
| ***Uncultured Streptococcus spp.*** | [GU145660.2](http://www.ncbi.nlm.nih.gov/nucleotide/323696495?report=genbank&log$=nucltop&blast_rank=4&RID=BGS136YC01S) | 8.61 | 100 |
| *Porphyromonas levii* | [AB547664.1](http://www.ncbi.nlm.nih.gov/nucleotide/302129302?report=genbank&log$=nucltop&blast_rank=1&RID=BGS136YC01S) | 6.62 | 100 |
| *Caulobacter leidyia* | [GQ891705.1](http://www.ncbi.nlm.nih.gov/nucleotide/260066246?report=genbank&log$=nucltop&blast_rank=6&RID=BGS136YC01S) | 3.97 | 100 |
| *Uncultured Bacteroides spp.* | [EU289070.1](http://www.ncbi.nlm.nih.gov/nucleotide/162296227?report=genbank&log$=nucltop&blast_rank=10&RID=BGS136YC01S) | 3.97 | 100 |
| *Uncultured bacterium* | [GQ179047.1](http://www.ncbi.nlm.nih.gov/nucleotide/283485014?report=genbank&log$=nucltop&blast_rank=1&RID=BGS136YC01S) | 3.31 | 98 |
| *Geobacillus pallidus* | [HM030740.1](http://www.ncbi.nlm.nih.gov/nucleotide/295853594?report=genbank&log$=nucltop&blast_rank=4&RID=BGS136YC01S) | 2.65 | 99 |
| *Uncultured bacterium* | [AM183009.1](http://www.ncbi.nlm.nih.gov/nucleotide/157690463?report=genbank&log$=nucltop&blast_rank=1&RID=BGS136YC01S) | 1.99 | 95 |
| *Uncultured Bacteroidetes bacterium* | [GU954712.1](http://www.ncbi.nlm.nih.gov/nucleotide/291328180?report=genbank&log$=nucltop&blast_rank=2&RID=BGS136YC01S) | 1.99 | 99 |
| *Uncultured bacterium* | [HM318928.1](http://www.ncbi.nlm.nih.gov/nucleotide/297012523?report=genbank&log$=nucltop&blast_rank=1&RID=BGS136YC01S) | 1.32 | 95 |
| *Uncultured bacterium* | [EU290135.1](http://www.ncbi.nlm.nih.gov/nucleotide/167595726?report=genbank&log$=nucltop&blast_rank=1&RID=BGS136YC01S) | 1.32 | 99 |
| *Uncultured bacterium* | [EU458333.1](http://www.ncbi.nlm.nih.gov/nucleotide/169273808?report=genbank&log$=nucltop&blast_rank=1&RID=BGS136YC01S) | 1.32 | 99 |
| *Staphylococcus equorum subsp. linens* | [NR_041926.1](http://www.ncbi.nlm.nih.gov/nucleotide/343198492?report=genbank&log$=nucltop&blast_rank=10&RID=BGS136YC01S) | 1.32 | 100 |
| *Uncultured bacterium* | [HQ420215.1](http://www.ncbi.nlm.nih.gov/nucleotide/312142133?report=genbank&log$=nucltop&blast_rank=1&RID=BGS136YC01S) | 1.32 | 99 |
| *Paenibacillus borealis* | [HM563046.1](http://www.ncbi.nlm.nih.gov/nucleotide/302035379?report=genbank&log$=nucltop&blast_rank=1&RID=BGS136YC01S) | 1.32 | 98 |
| *Ureaplasma diversum* | [NR_025878.1](http://www.ncbi.nlm.nih.gov/nucleotide/219846288?report=genbank&log$=nucltop&blast_rank=1&RID=BGS136YC01S) | 0.66 | 99 |
| *Uncultured Ruminococcaceae bacterium* | [EU794228.1](http://www.ncbi.nlm.nih.gov/nucleotide/192792279?report=genbank&log$=nucltop&blast_rank=1&RID=BGS136YC01S) | 0.66 | 99 |
| *Thermoanaerobacterales bacterium* | [GU797851.1](http://www.ncbi.nlm.nih.gov/nucleotide/293509135?report=genbank&log$=nucltop&blast_rank=6&RID=BGS136YC01S) | 0.66 | 99 |
| *Bacillus firmus* | [EF636895.1](http://www.ncbi.nlm.nih.gov/nucleotide/157057246?report=genbank&log$=nucltop&blast_rank=1&RID=BGS136YC01S) | 0.66 | 100 |
| *Swine manure pit bacterium* | [AF445295.1](http://www.ncbi.nlm.nih.gov/nucleotide/17940546?report=genbank&log$=nucltop&blast_rank=1&RID=BGS136YC01S) | 0.66 | 99 |
| *Uncultured bacterium* | [GU615111.1](http://www.ncbi.nlm.nih.gov/nucleotide/290601705?report=genbank&log$=nucltop&blast_rank=1&RID=BGS136YC01S) | 0.66 | 99 |
| *Uncultured bacterium* | [FN994146.1](http://www.ncbi.nlm.nih.gov/nucleotide/304656162?report=genbank&log$=nucltop&blast_rank=1&RID=BGS136YC01S) | 0.66 | 99 |
| *Arthrobacter spp.* | [AM260537.1](http://www.ncbi.nlm.nih.gov/nucleotide/107593744?report=genbank&log$=nucltop&blast_rank=7&RID=BGS136YC01S) | 0.66 | 100 |
| *Uncultured bacterium* | [JF553154.1](http://www.ncbi.nlm.nih.gov/nucleotide/341988339?report=genbank&log$=nucltop&blast_rank=1&RID=BGS136YC01S) | 0.66 | 94 |
| *Uncultured bacterium* | [GU609709.1](http://www.ncbi.nlm.nih.gov/nucleotide/290596302?report=genbank&log$=nucltop&blast_rank=1&RID=BGS136YC01S) | 0.66 | 99 |
| *Uncultured bacterium* | [AY816889.1](http://www.ncbi.nlm.nih.gov/nucleotide/55831820?report=genbank&log$=nucltop&blast_rank=1&RID=BGS136YC01S) | 0.66 | 87 |
| *Uncultured bacterium* | [HM327761.1](http://www.ncbi.nlm.nih.gov/nucleotide/297021356?report=genbank&log$=nucltop&blast_rank=1&RID=BGS136YC01S) | 0.66 | 85 |
| *Uncultured bacterium* | [EU290118.1](http://www.ncbi.nlm.nih.gov/nucleotide/167595709?report=genbank&log$=nucltop&blast_rank=1&RID=BGS136YC01S) | 0.66 | 100 |
| *Uncultured bacterium* | [AB200302.1](http://www.ncbi.nlm.nih.gov/nucleotide/60360943?report=genbank&log$=nucltop&blast_rank=1&RID=BGS136YC01S) | 0.66 | 95 |
| *Uncultured bacterium* | [GU613627.1](http://www.ncbi.nlm.nih.gov/nucleotide/290600221?report=genbank&log$=nucltop&blast_rank=1&RID=BGS136YC01S) | 0.66 | 99 |
| *Uncultured bacterium* | [FJ368239.1](http://www.ncbi.nlm.nih.gov/nucleotide/214022623?report=genbank&log$=nucltop&blast_rank=1&RID=BGS136YC01S) | 0.66 | 98 |
| *Uncultured bacterium* | [JF643239.1](http://www.ncbi.nlm.nih.gov/nucleotide/342078424?report=genbank&log$=nucltop&blast_rank=1&RID=BGS136YC01S) | 0.66 | 99 |
| *Clostridium perfringens* | [AB627081.1](http://www.ncbi.nlm.nih.gov/nucleotide/332144243?report=genbank&log$=nucltop&blast_rank=1&RID=BGS136YC01S) | 0.66 | 99 |
| *Uncultured bacterium* | [EU773780.1](http://www.ncbi.nlm.nih.gov/nucleotide/192979757?report=genbank&log$=nucltop&blast_rank=1&RID=BGS136YC01S) | 0.66 | 96 |
| *Uncultured organism* | [JF782021.1](http://www.ncbi.nlm.nih.gov/nucleotide/333496362?report=genbank&log$=nucltop&blast_rank=1&RID=BGS136YC01S) | 0.66 | 97 |
| *Uncultured bacterium* | [DQ793282.1](http://www.ncbi.nlm.nih.gov/nucleotide/110433682?report=genbank&log$=nucltop&blast_rank=1&RID=BGS136YC01S) | 0.66 | 95 |
| *Uncultured bacterium* | [FJ682049.1](http://www.ncbi.nlm.nih.gov/nucleotide/223694926?report=genbank&log$=nucltop&blast_rank=1&RID=BGS136YC01S) | 0.66 | 99 |
| *Uncultured Prevotella spp.* | [GU905979.1](http://www.ncbi.nlm.nih.gov/nucleotide/294613821?report=genbank&log$=nucltop&blast_rank=1&RID=BGS136YC01S) | 0.66 | 98 |
| *Clostridium ramosum* | [AB595128.1](http://www.ncbi.nlm.nih.gov/nucleotide/309710775?report=genbank&log$=nucltop&blast_rank=5&RID=BGS136YC01S) | 0.66 | 100 |
| *Uncultured bacterium* | [HQ319439.1](http://www.ncbi.nlm.nih.gov/nucleotide/310719631?report=genbank&log$=nucltop&blast_rank=10&RID=BGS136YC01S) | 0.66 | 94 |
| *Uncultured bacterium* | [GU615040.1](http://www.ncbi.nlm.nih.gov/nucleotide/290601634?report=genbank&log$=nucltop&blast_rank=1&RID=BGS136YC01S) | 0.66 | 91 |
| *Uncultured bacterium* | [GU609321.1](http://www.ncbi.nlm.nih.gov/nucleotide/290595914?report=genbank&log$=nucltop&blast_rank=1&RID=BGS136YC01S) | 0.66 | 95 |
| *Uncultured bacterium* | [FJ507484.1](http://www.ncbi.nlm.nih.gov/nucleotide/219530679?report=genbank&log$=nucltop&blast_rank=1&RID=BGS136YC01S) | 0.66 | 99 |
| *Halomonas spp.* | [AJ302088.1](http://www.ncbi.nlm.nih.gov/nucleotide/12697323?report=genbank&log$=nucltop&blast_rank=1&RID=BGS136YC01S) | 0.66 | 100 |
