# Supplementary material for: Microbial Diversity of Bovine Mastitic Milk as Described by Pyrosequencing of Metagenomic 16s rDNA
Source: PLoS One. 2012 Oct 17;7(10):e47671. doi: 10.1371/journal.pone.0047671 (PMC3474744; doi:10.1371/journal.pone.0047671)
Supplement: Table S9 — Species level information (with GenBank Accession number, and identity match) for the predominant representative sequences in samples characterized as Streptococcus uberis mastitis. (DOCX) [file pone.0047671.s009.docx]

| Species | Accession No | Prevalence | Identity (%) |
| --- | --- | --- | --- |
| ***Streptococcus uberis*** | [HQ326695.1](http://www.ncbi.nlm.nih.gov/nucleotide/308390715?report=genbank&log$=nucltop&blast_rank=6&RID=BGJFVK5101S) | 34.84 | 100 |
| *Caulobacter leidyia* | [GQ891705.1](http://www.ncbi.nlm.nih.gov/nucleotide/260066246?report=genbank&log$=nucltop&blast_rank=6&RID=BGJFVK5101S) | 5.11 | 100 |
| *Porphyromonas levii* | [AB547664.1](http://www.ncbi.nlm.nih.gov/nucleotide/302129302?report=genbank&log$=nucltop&blast_rank=1&RID=BGJFVK5101S) | 4.77 | 100 |
| *Fusobacterium necrophorum subsp. Funduliforme* | [AB525413.1](http://www.ncbi.nlm.nih.gov/nucleotide/261228522?report=genbank&log$=nucltop&blast_rank=5&RID=BGJFVK5101S) | 3.50 | 100 |
| *Uncultured Porphyromonas spp.* | [HM754526.1](http://www.ncbi.nlm.nih.gov/nucleotide/304365992?report=genbank&log$=nucltop&blast_rank=1&RID=BGJFVK5101S) | 3.16 | 100 |
| *Uncultured bacterium* | [AM183009.1](http://www.ncbi.nlm.nih.gov/nucleotide/157690463?report=genbank&log$=nucltop&blast_rank=1&RID=BGJFVK5101S) | 2.89 | 95 |
| *Geobacillus pallidus* | [HM030740.1](http://www.ncbi.nlm.nih.gov/nucleotide/295853594?report=genbank&log$=nucltop&blast_rank=6&RID=BGJFVK5101S) | 2.69 | 99 |
| *Uncultured Porphyromonas spp.* | [HM754526.1](http://www.ncbi.nlm.nih.gov/nucleotide/304365992?report=genbank&log$=nucltop&blast_rank=1&RID=BGJFVK5101S) | 2.62 | 99 |
| *Uncultured bacterium* | [JF643239.1](http://www.ncbi.nlm.nih.gov/nucleotide/342078424?report=genbank&log$=nucltop&blast_rank=1&RID=BGJFVK5101S) | 2.22 | 100 |
| *Uncultured bacterium* | [JF663845.1](http://www.ncbi.nlm.nih.gov/nucleotide/342099030?report=genbank&log$=nucltop&blast_rank=1&RID=BGJFVK5101S) | 1.88 | 98 |
| *Rumen bacterium enrichment culture* | [HM597702.1](http://www.ncbi.nlm.nih.gov/nucleotide/304569903?report=genbank&log$=nucltop&blast_rank=1&RID=BGJFVK5101S) | 1.61 | 100 |
| *Uncultured bacterium* | [HM318928.1](http://www.ncbi.nlm.nih.gov/nucleotide/297012523?report=genbank&log$=nucltop&blast_rank=1&RID=BGJFVK5101S) | 1.41 | 94 |
| *Propionibacterium acnes* | [CP003084.1](http://www.ncbi.nlm.nih.gov/nucleotide/353454017?report=genbank&log$=nucltop&blast_rank=1&RID=BGJFVK5101S) | 1.08 | 100 |
| *Staphylococcus equorum subsp. linens* | [NR_041926.1](http://www.ncbi.nlm.nih.gov/nucleotide/343198492?report=genbank&log$=nucltop&blast_rank=10&RID=BGJFVK5101S) | 1.08 | 100 |
| *Bacteroides heparinolyticus* | [GQ422742.1](http://www.ncbi.nlm.nih.gov/nucleotide/257480655?report=genbank&log$=nucltop&blast_rank=3&RID=BGJFVK5101S) | 0.81 | 100 |
| *Swine manure pit bacterium* | [AF445295.1](http://www.ncbi.nlm.nih.gov/nucleotide/17940546?report=genbank&log$=nucltop&blast_rank=1&RID=BGJFVK5101S) | 0.81 | 99 |
| *Uncultured bacterium* | [FJ682454.1](http://www.ncbi.nlm.nih.gov/nucleotide/223695331?report=genbank&log$=nucltop&blast_rank=1&RID=BGJFVK5101S) | 0.67 | 99 |
| *Uncultured bacterium* | [EU290118.1](http://www.ncbi.nlm.nih.gov/nucleotide/167595709?report=genbank&log$=nucltop&blast_rank=1&RID=BGJFVK5101S) | 0.67 | 100 |
| *Uncultured bacterium* | [EU458333.1](http://www.ncbi.nlm.nih.gov/nucleotide/169273808?report=genbank&log$=nucltop&blast_rank=1&RID=BGJFVK5101S) | 0.67 | 98 |
| *Prevotella spp.* | [FJ848548.1](http://www.ncbi.nlm.nih.gov/nucleotide/225733529?report=genbank&log$=nucltop&blast_rank=5&RID=BGJFVK5101S) | 0.54 | 100 |
| *Peptostreptococcus anaerobius* | [AB640695.1](http://www.ncbi.nlm.nih.gov/nucleotide/336454741?report=genbank&log$=nucltop&blast_rank=1&RID=BGJFVK5101S) | 0.54 | 98 |
| *Helcococcus ovis* | [AB542088.1](http://www.ncbi.nlm.nih.gov/nucleotide/284049428?report=genbank&log$=nucltop&blast_rank=10&RID=BGJFVK5101S) | 0.47 | 100 |
| *Uncultured Prevotella spp.* | [GU905979.1](http://www.ncbi.nlm.nih.gov/nucleotide/294613821?report=genbank&log$=nucltop&blast_rank=1&RID=BGJFVK5101S) | 0.47 | 98 |
| *Staphylococcus epidermidis* | [JN644588.1](http://www.ncbi.nlm.nih.gov/nucleotide/348161500?report=genbank&log$=nucltop&blast_rank=1&RID=BGJFVK5101S) | 0.40 | 100 |
| *Corynebacterium falsenii* | [AF537594.1](http://www.ncbi.nlm.nih.gov/nucleotide/23954564?report=genbank&log$=nucltop&blast_rank=2&RID=BGJFVK5101S) | 0.40 | 100 |
| *Ochrobactrum pseudogrignonense* | [FJ859687.2](http://www.ncbi.nlm.nih.gov/nucleotide/272825711?report=genbank&log$=nucltop&blast_rank=1&RID=BGJFVK5101S) | 0.40 | 99 |
| *Halomonas spp.* | [AJ302088.1](http://www.ncbi.nlm.nih.gov/nucleotide/12697323?report=genbank&log$=nucltop&blast_rank=1&RID=BGJFVK5101S) | 0.34 | 99 |
| *Bacteroides vulgatus* | [JN084208.1](http://www.ncbi.nlm.nih.gov/nucleotide/341832603?report=genbank&log$=nucltop&blast_rank=2&RID=BGJFVK5101S) | 0.34 | 100 |
| *Uncultured bacterium* | [GU603837.1](http://www.ncbi.nlm.nih.gov/nucleotide/290590430?report=genbank&log$=nucltop&blast_rank=1&RID=BGJFVK5101S) | 0.34 | 98 |
| *Ureaplasma diversum* | [NR_025878.1](http://www.ncbi.nlm.nih.gov/nucleotide/219846288?report=genbank&log$=nucltop&blast_rank=1&RID=BGJFVK5101S) | 0.34 | 98 |
| *Paenibacillus borealis* | [HM563046.1](http://www.ncbi.nlm.nih.gov/nucleotide/302035379?report=genbank&log$=nucltop&blast_rank=1&RID=BGJFVK5101S) | 0.34 | 99 |
