# Supplementary material for: Microbial Diversity of Bovine Mastitic Milk as Described by Pyrosequencing of Metagenomic 16s rDNA
Source: PLoS One. 2012 Oct 17;7(10):e47671. doi: 10.1371/journal.pone.0047671 (PMC3474744; doi:10.1371/journal.pone.0047671)
Supplement: Table S10 — Species level information (with GenBank Accession number, and identity match) for the predominant representative sequences in samples obtained from healthy cows and had a low SCC. (DOCX) [file pone.0047671.s010.docx]

| Species | Asseccion No | Prevalence | Identity (%) |
| --- | --- | --- | --- |
| *Uncultured Propionibacterium sp.* | [JQ288555.1](http://www.ncbi.nlm.nih.gov/nucleotide/383615439?report=genbank&log$=nucltop&blast_rank=10&RID=1SYPJ77V01N) | 14.00 | 100 |
| *Uncultured bacterium* | [EF205691.1](http://www.ncbi.nlm.nih.gov/nucleotide/146285427?report=genbank&log$=nucltop&blast_rank=3&RID=1SYPJ77V01N) | 8.03 | 100 |
| *Staphylococcus epidermidis* | [JQ795860.1](http://www.ncbi.nlm.nih.gov/nucleotide/387287106?report=genbank&log$=nucltop&blast_rank=3&RID=1X1N60ZW01N) | 2.07 | 100 |
| *Uncultured proteobacterium* | [GU956128.1](http://www.ncbi.nlm.nih.gov/nucleotide/291329596?report=genbank&log$=nucltop&blast_rank=2&RID=1X1N60ZW01N) | 2.07 | 100 |
| *Uncultured bacterium* | [GU634642.1](http://www.ncbi.nlm.nih.gov/nucleotide/290621237?report=genbank&log$=nucltop&blast_rank=4&RID=1X1N60ZW01N) | 1.99 | 99 |
| *Bacteroides heparinolyticus* | [GQ422742.1](http://www.ncbi.nlm.nih.gov/nucleotide/257480655?report=genbank&log$=nucltop&blast_rank=6&RID=1X1N60ZW01N) | 1.84 | 99 |
| *Frateuria sp.* | [AY495959.1](http://www.ncbi.nlm.nih.gov/nucleotide/40549113?report=genbank&log$=nucltop&blast_rank=8&RID=1X1N60ZW01N) | 1.61 | 99 |
| *Uncultured bacterium* | [FJ682300.1](http://www.ncbi.nlm.nih.gov/nucleotide/223695177?report=genbank&log$=nucltop&blast_rank=1&RID=1X1N60ZW01N) | 1.53 | 100 |
| *Streptococcus uberis* | [HQ326695.1](http://www.ncbi.nlm.nih.gov/nucleotide/308390715?report=genbank&log$=nucltop&blast_rank=6&RID=1X1N60ZW01N) | 1.53 | 100 |
| *Uncultured bacterium* | [AY511727.1](http://www.ncbi.nlm.nih.gov/nucleotide/40890946?report=genbank&log$=nucltop&blast_rank=4&RID=1X1N60ZW01N) | 1.38 | 100 |
| *Bacteroides vulgatus* | [JN084208.1](http://www.ncbi.nlm.nih.gov/nucleotide/341832603?report=genbank&log$=nucltop&blast_rank=5&RID=1X1N60ZW01N) | 1.15 | 100 |
| *Uncultured Bacteroides sp.* | [JQ083405.1](http://www.ncbi.nlm.nih.gov/nucleotide/374094719?report=genbank&log$=nucltop&blast_rank=1&RID=1X1N60ZW01N) | 1.07 | 100 |
| *Uncultured bacterium* | [EU772991.1](http://www.ncbi.nlm.nih.gov/nucleotide/192978968?report=genbank&log$=nucltop&blast_rank=1&RID=1X1N60ZW01N) | 0.99 | 99 |
| *Uncultured bacterium* | [EU290137.1](http://www.ncbi.nlm.nih.gov/nucleotide/167595728?report=genbank&log$=nucltop&blast_rank=1&RID=1X1N60ZW01N) | 0.99 | 99 |
| *Bacteroides fragilis* | [AB618792.1](http://www.ncbi.nlm.nih.gov/nucleotide/325651810?report=genbank&log$=nucltop&blast_rank=7&RID=1X1N60ZW01N) | 0.84 | 99 |
| *Uncultured Porphyromonas sp.* | [JN167617.1](http://www.ncbi.nlm.nih.gov/nucleotide/347547330?report=genbank&log$=nucltop&blast_rank=2&RID=1X1N60ZW01N) | 0.84 | 99 |
| *Uncultured bacterium* | [JX108076.1](http://www.ncbi.nlm.nih.gov/nucleotide/395480040?report=genbank&log$=nucltop&blast_rank=1&RID=1X1N60ZW01N) | 0.84 | 99 |
| *Rhodanobacter terrae* | [FJ405366.1](http://www.ncbi.nlm.nih.gov/nucleotide/212291434?report=genbank&log$=nucltop&blast_rank=3&RID=1X1N60ZW01N) | 0.61 | 99 |
| *Uncultured bacterium* | [JX108142.1](http://www.ncbi.nlm.nih.gov/nucleotide/395480106?report=genbank&log$=nucltop&blast_rank=1&RID=1X1N60ZW01N) | 0.61 | 100 |
| *Pelistega europaea* | [FJ999734.1](http://www.ncbi.nlm.nih.gov/nucleotide/289468036?report=genbank&log$=nucltop&blast_rank=2&RID=1X1N60ZW01N) | 0.61 | 98 |
| *Uncultured bacterium* | [JX108447.1](http://www.ncbi.nlm.nih.gov/nucleotide/395480411?report=genbank&log$=nucltop&blast_rank=1&RID=1X1N60ZW01N) | 0.61 | 99 |
| *Clostridiales bacterium* | [HQ452852.1](http://www.ncbi.nlm.nih.gov/nucleotide/312064779?report=genbank&log$=nucltop&blast_rank=1&RID=1X1N60ZW01N) | 0.61 | 100 |
| *Uncultured bacterium* | [JF086910.1](http://www.ncbi.nlm.nih.gov/nucleotide/321963614?report=genbank&log$=nucltop&blast_rank=1&RID=1X1N60ZW01N) | 0.54 | 100 |
| *Uncultured bacterium* | [FN658975.1](http://www.ncbi.nlm.nih.gov/nucleotide/335334410?report=genbank&log$=nucltop&blast_rank=1&RID=1X1N60ZW01N) | 0.54 | 100 |
| *Uncultured bacterium* | [JX013206.1](http://www.ncbi.nlm.nih.gov/nucleotide/392329302?report=genbank&log$=nucltop&blast_rank=1&RID=1X1N60ZW01N) | 0.54 | 100 |
| *Staphylococcus equorum* | [JN969599.1](http://www.ncbi.nlm.nih.gov/nucleotide/378747664?report=genbank&log$=nucltop&blast_rank=2&RID=1X1N60ZW01N) | 0.54 | 100 |
| *Uncultured bacterium* | [JF194563.1](http://www.ncbi.nlm.nih.gov/nucleotide/322179968?report=genbank&log$=nucltop&blast_rank=1&RID=1X1N60ZW01N) | 0.46 | 100 |
| *Uncultured Corynebacterium sp.* | [JN584700.1](http://www.ncbi.nlm.nih.gov/nucleotide/379334222?report=genbank&log$=nucltop&blast_rank=4&RID=1X1N60ZW01N) | 0.46 | 100 |
| *Uncultured Helcococcus sp.* | [JN167606.1](http://www.ncbi.nlm.nih.gov/nucleotide/347547319?report=genbank&log$=nucltop&blast_rank=2&RID=1X1N60ZW01N) | 0.46 | 100 |
| *Clostridium sp.* | [AB739698.1](http://www.ncbi.nlm.nih.gov/nucleotide/399219965?report=genbank&log$=nucltop&blast_rank=2&RID=1X1N60ZW01N) | 0.46 | 99 |
| *Uncultured bacterium* | [EF205694.1](http://www.ncbi.nlm.nih.gov/nucleotide/146285430?report=genbank&log$=nucltop&blast_rank=1&RID=1X1N60ZW01N) | 0.46 | 99 |
| *Uncultured bacterium* | [JX106677.1](http://www.ncbi.nlm.nih.gov/nucleotide/395478641?report=genbank&log$=nucltop&blast_rank=1&RID=1X1N60ZW01N) | 0.46 | 99 |
| *Uncultured bacterium* | [FN658980.1](http://www.ncbi.nlm.nih.gov/nucleotide/335334415?report=genbank&log$=nucltop&blast_rank=1&RID=1X1N60ZW01N) | 0.46 | 100 |
| *Uncultured Clostridiales bacterium* | [HM076511.1](http://www.ncbi.nlm.nih.gov/nucleotide/297351348?report=genbank&log$=nucltop&blast_rank=10&RID=1X1N60ZW01N) | 0.46 | 100 |
| *Uncultured Bacteroides sp.* | [JN167633.1](http://www.ncbi.nlm.nih.gov/nucleotide/347547346?report=genbank&log$=nucltop&blast_rank=2&RID=1X1N60ZW01N) | 0.46 | 100 |
| *Uncultured Firmicutes bacterium* | [HE583207.1](http://www.ncbi.nlm.nih.gov/nucleotide/348605886?report=genbank&log$=nucltop&blast_rank=1&RID=1X1N60ZW01N) | 0.46 | 100 |
| *Uncultured bacterium clone* | [AY511727.1](http://www.ncbi.nlm.nih.gov/nucleotide/40890946?report=genbank&log$=nucltop&blast_rank=4&RID=1XP99XZP01S) | 0.46 | 100 |
| *Uncultured bacterium clone* | [EU474921.1](http://www.ncbi.nlm.nih.gov/nucleotide/169290396?report=genbank&log$=nucltop&blast_rank=1&RID=1XP99XZP01S) | 0.46 | 97 |
| *Uncultured bacterium* | [JX107110.1](http://www.ncbi.nlm.nih.gov/nucleotide/395479074?report=genbank&log$=nucltop&blast_rank=1&RID=1XP99XZP01S) | 0.38 | 99 |
| *Propionibacterium granulosum* | [AB638444.1](http://www.ncbi.nlm.nih.gov/nucleotide/335334839?report=genbank&log$=nucltop&blast_rank=3&RID=1XP99XZP01S) | 0.38 | 100 |
| *Trueperella pyogenes* | [JN578141.1](http://www.ncbi.nlm.nih.gov/nucleotide/345847796?report=genbank&log$=nucltop&blast_rank=3&RID=1XP99XZP01S) | 0.38 | 100 |
| *Uncultured bacterium* | [JF137212.1](http://www.ncbi.nlm.nih.gov/nucleotide/322112963?report=genbank&log$=nucltop&blast_rank=1&RID=1XP99XZP01S) | 0.38 | 100 |
| *Uncultured Lachnospiraceae bacterium* | [EU794239.1](http://www.ncbi.nlm.nih.gov/nucleotide/192792290?report=genbank&log$=nucltop&blast_rank=2&RID=1XP99XZP01S) | 0.38 | 100 |
| *Uncultured bacterium* | [JX108364.1](http://www.ncbi.nlm.nih.gov/nucleotide/395480328?report=genbank&log$=nucltop&blast_rank=1&RID=1XP99XZP01S) | 0.38 | 99 |
| *Uncultured Porphyromonas sp.* | [HM754526.1](http://www.ncbi.nlm.nih.gov/nucleotide/304365992?report=genbank&log$=nucltop&blast_rank=2&RID=1XP99XZP01S) | 0.38 | 99 |
| *Uncultured bacterium* | [EU464106.1](http://www.ncbi.nlm.nih.gov/nucleotide/169279581?report=genbank&log$=nucltop&blast_rank=1&RID=1XP99XZP01S) | 0.38 | 99 |
| *Uncultured bacterium* | [GQ136816.1](http://www.ncbi.nlm.nih.gov/nucleotide/253766709?report=genbank&log$=nucltop&blast_rank=1&RID=1XP99XZP01S) | 0.38 | 99 |
| *Caulobacter leidyia* | [GQ891705.1](http://www.ncbi.nlm.nih.gov/nucleotide/260066246?report=genbank&log$=nucltop&blast_rank=8&RID=1XP99XZP01S) | 0.38 | 100 |
| *Halomonas sp.* | [AJ302088.1](http://www.ncbi.nlm.nih.gov/nucleotide/12697323?report=genbank&log$=nucltop&blast_rank=1&RID=1XP99XZP01S) | 0.31 | 100 |
| *Uncultured Lachnospiraceae bacterium* | [EF698785.1](http://www.ncbi.nlm.nih.gov/nucleotide/154191551?report=genbank&log$=nucltop&blast_rank=1&RID=1XP99XZP01S) | 0.31 | 99 |
| *Uncultured Clostridiales bacterium* | [JQ083415.1](http://www.ncbi.nlm.nih.gov/nucleotide/374094729?report=genbank&log$=nucltop&blast_rank=1&RID=1XP99XZP01S) | 0.31 | 99 |
| *Uncultured bacterium* | [JX108113.1](http://www.ncbi.nlm.nih.gov/nucleotide/395480077?report=genbank&log$=nucltop&blast_rank=1&RID=1XP99XZP01S) | 0.31 | 100 |
| *Ornithinibacillus sp.* | [GQ903473.1](http://www.ncbi.nlm.nih.gov/nucleotide/260842115?report=genbank&log$=nucltop&blast_rank=1&RID=1XP99XZP01S) | 0.31 | 100 |
| *Uncultured bacterium* | [HM339654.1](http://www.ncbi.nlm.nih.gov/nucleotide/297033249?report=genbank&log$=nucltop&blast_rank=1&RID=1XP99XZP01S) | 0.31 | 99 |
| *Uncultured bacterium* | [JX120473.1](http://www.ncbi.nlm.nih.gov/nucleotide/395550130?report=genbank&log$=nucltop&blast_rank=1&RID=1XP99XZP01S) | 0.31 | 100 |
| *Geobacillus tepidamans* | [FJ823100.2](http://www.ncbi.nlm.nih.gov/nucleotide/345106913?report=genbank&log$=nucltop&blast_rank=1&RID=1XP99XZP01S) | 0.31 | 99 |
| *Uncultured bacterium* | [JX087346.1](http://www.ncbi.nlm.nih.gov/nucleotide/389483870?report=genbank&log$=nucltop&blast_rank=1&RID=1ZAK2JU601N) | 0.31 | 100 |
| *Uncultured bacterium* | [JX108142.1](http://www.ncbi.nlm.nih.gov/nucleotide/395480106?report=genbank&log$=nucltop&blast_rank=1&RID=1ZAK2JU601N) | 0.31 | 99 |
| *Uncultured Lactobacillus* | [JQ083429.1](http://www.ncbi.nlm.nih.gov/nucleotide/374094743?report=genbank&log$=nucltop&blast_rank=3&RID=1ZAK2JU601N) | 0.31 | 100 |
| *Uncultured bacterium clone* | [GQ448802.1](http://www.ncbi.nlm.nih.gov/nucleotide/258548456?report=genbank&log$=nucltop&blast_rank=1&RID=1ZAK2JU601N) | 0.31 | 100 |
| *Flavobacterium sp.* | [HQ836451.1](http://www.ncbi.nlm.nih.gov/nucleotide/323126135?report=genbank&log$=nucltop&blast_rank=10&RID=1ZAK2JU601N) | 0.31 | 100 |
| *Uncultured bacterium* | [JN834156.1](http://www.ncbi.nlm.nih.gov/nucleotide/358250462?report=genbank&log$=nucltop&blast_rank=1&RID=1ZAK2JU601N) | 0.31 | 98 |
| *Uncultured bacterium* | [JQ825064.1](http://www.ncbi.nlm.nih.gov/nucleotide/386872321?report=genbank&log$=nucltop&blast_rank=1&RID=1ZAK2JU601N) | 0.31 | 92 |
| *Uncultured organism* | [HQ766342.1](http://www.ncbi.nlm.nih.gov/nucleotide/319473481?report=genbank&log$=nucltop&blast_rank=1&RID=1ZAK2JU601N) | 0.31 | 100 |
| *Uncultured Firmicutes bacterium* | [HE583206.1](http://www.ncbi.nlm.nih.gov/nucleotide/348605885?report=genbank&log$=nucltop&blast_rank=8&RID=1ZAK2JU601N) | 0.31 | 100 |
